# Maternal and Peri/Neonatal Mortality and Morbidity in Midwife‐Assisted Low‐Risk Birth Outside Hospital Compared to Obstetric Team‐Assisted Low‐Risk Hospital Birth: A Systematic Review and Meta‐Analysis

**DOI:** 10.1111/apa.70685

**Published:** 2026-07-20

**Authors:** Carolina Herding, Ulla‐Britt Wennerholm, Anders Elfvin, Sara Holm, Karolina Linden, Jahangir Khan, Max Petzold, Christina Bergh, Petteri Sjögren, Ida Stadig, Ann Liljegren, Ylva Carlsson, Verena Sengpiel

**Affiliations:** ^1^ Department of Obstetrics and Gynecology Sahlgrenska University Hospital Gothenburg Sweden; ^2^ Department of Obstetrics and Gynecology, Institute of Clinical Sciences, Sahlgrenska Academy University of Gothenburg Gothenburg Sweden; ^3^ Department of Pediatrics, Institute of Clinical Sciences, Sahlgrenska Academy University of Gothenburg Gothenburg Sweden; ^4^ Department of Pediatrics, The Queen Silvia Children's Hospital Sahlgrenska University Hospital Gothenburg Sweden; ^5^ Institute of Health and Care Sciences, Sahlgrenska Academy University of Gothenburg Gothenburg Sweden; ^6^ Centre for Health Technology Assessment Sahlgrenska University Hospital Gothenburg Sweden; ^7^ School of Public Health and Community Medicine, Institute of Medicine University of Gothenburg Gothenburg Sweden; ^8^ Department of International Public Health Liverpool School of Tropical Medicine Liverpool UK; ^9^ Medical Library Sahlgrenska University Hospital Gothenburg Sweden

**Keywords:** freestanding midwifery unit, health economics, home birth, maternal morbidity, maternal mortality, neonatal morbidity, neonatal mortality, perinatal mortality

## Abstract

**Aim:**

To compare outcomes of planned home or freestanding midwifery unit (FMU) with obstetric unit births in high‐income countries.

**Methods:**

We conducted a systematic review and meta‐analysis of systematic reviews, randomised controlled trials, cohort studies, case series, and case reports. Medline, Embase, CINAHL and the Cochrane Library were searched up to 8 April 2026 (PROSPERO: CRD42024570558). Outcomes included maternal and peri/neonatal mortality, severe morbidity, hospital transfers and health economics. Certainty of evidence was assessed using GRADE.

**Results:**

Nine cohort studies (*n* = 1 261 312), 18 case series, and three case reports were included. All cohort studies had a risk of bias. Planned home/FMU births were associated with fewer maternal intensive care admissions (adjusted odds ratio [AOR] 0.41, 95% confidence interval [95% CI] 0.19–0.89), increased peri/neonatal mortality (OR 1.70, 95% CI 1.05–2.74), fewer severe perineal tears (AOR 0.64, 95% CI 0.45–0.93), and lower intrapartum caesarean section rates (AOR 0.29, 95% CI 0.26–0.33). Transfers occurred in 9%–33% of births. Cost savings for home/FMU births disappeared when emergency standby capacity was considered.

**Conclusion:**

Evidence certainty was low or very low. Planned home/FMU births may reduce some maternal complications but may increase peri/neonatal mortality, although absolute risks remain low. The cost‐effectiveness of home/FMU birth remains uncertain.

## Summary

- Planned home and freestanding midwifery unit (FMU) births remain controversial, and robust evidence on maternal and peri/neonatal safety is needed to inform maternity care decisions.
- This systematic review found fewer maternal complications but increased peri/neonatal mortality with planned home/FMU births although absolute risks were low.
- The certainty of evidence was low or very low, highlighting the need for further research and careful counselling about potential benefits, risks and service requirements.

**Abbreviations**
AMU: alongside midwifery unit
AOR: adjusted odds ratio
FMU: freestanding midwifery unit
HIE: hypoxic–ischemic encephalopathy
HTA: Health Technology Assessment
ICM: International Confederation of Midwives
NICU: admission to neonatal ward or neonatal intensive care unit
OR: odds ratio

## 1 Introduction

In some countries, planned out‐of‐hospital births, including home births and freestanding midwifery unit (FMU) births, are offered as alternatives to hospital‐based care for women with expected low‐risk births. These settings have been associated with fewer obstetric interventions and higher maternal satisfaction in some studies, reflecting preferences for autonomy, continuity of care, and avoidance of unnecessary medicalisation [1, 2].

Concerns remain regarding neonatal outcomes in planned out‐of‐hospital births, particularly peri/neonatal mortality. Although childbirth is a physiological process, it carries inherent risks that may require urgent obstetric and neonatal interventions, even in expected low‐risk births. Observational studies have reported conflicting results, with some studies suggesting comparable safety to hospital births, while others—particularly large population‐based cohort studies—have reported higher peri/neonatal mortality in planned out‐of‐hospital births, especially among nulliparous women [3, 4].

Rates of planned out‐of‐hospital births vary substantially across high‐income countries. The Netherlands is a notable exception, with a historically high prevalence of planned home births [5]. In most other high‐income settings, planned home births remain uncommon and have been studied mostly within publicly funded maternity care systems with formal integration of out‐of‐hospital births [6]. In Australia, planned home births are rare, whereas FMUs are an established alternative model of care in some regions [4]. Overall, international variations in out‐of‐hospital births reflect differences in the organisation, regulation, and integration of maternity care services rather than maternal risk profiles alone [6].

Previous reviews pooled data across diverse healthcare systems and varying low‐risk definitions, which limited their applicability to strictly defined low‐risk populations [6, 7, 8]. Importantly, several cohort studies contributing to these reviews used hospital comparison groups that included midwife‐led hospital settings, such as alongside midwifery units (AMU), rather than obstetric units exclusively. Further, only some reviews, including Hutton [7] and Reitsma [8], presented stratified analyses by parity. Given these limitations, uncertainty persists regarding the safety of planned out‐of‐hospital births for women with strictly defined expected low‐risk births in high‐income countries with midwifery education in accordance with the International Confederation of Midwives (ICM) [9] criteria compared to obstetric unit births. A rigorous synthesis of the available evidence is needed to clarify the maternal and peri/neonatal risks and benefits, thereby supporting informed decision‐making for women and guiding clinical practice and policy.

The objective of this systematic review and meta‐analysis was to compare maternal and peri/neonatal mortality and severe morbidity in planned midwife‐assisted home/FMU births with obstetric team‐assisted births at the hospital in strictly defined expected low‐risk births in high‐income countries with midwifery education in accordance with the ICM criteria.

## 2 Materials and Methods

### 2.1 Study Design

This systematic review is based on a Health Technology Assessment (HTA) report conducted by the authors in accordance with regional HTA guidelines, preregistered at the HTA‐centre, Sahlgrenska University Hospital, Gothenburg, Sweden (2025) including an updated literature search up to 8 April 2026. The HTA report is publicly available from the same institution [10]. The review was registered in the International Prospective Register of Systematic Reviews (PROSPERO; CRD42024570558 registered 2024‐10‐07) [11] before data extraction and was conducted in accordance with the Preferred Reporting Items for Systematic Reviews and Meta‐Analyses (PRISMA) 2020 guidelines [12]. Ethical approval was not required as no individual‐level data were collected or analysed.

### 2.2 Eligibility Criteria

The PICO (population‐intervention‐comparator‐outcome) was defined before the literature search commenced (Figure 1). Outcomes from core outcome sets were discussed [13], adapted to the objective and rated according to their importance for decision making as critical, important or less important. Eligible studies included systematic reviews, randomised controlled trials, and non‐randomised controlled studies with at least 100 participants comparing planned midwife‐assisted home/FMU births with planned obstetric unit births (e.g., excluding hospital birth at AMUs) among women with expected low‐risk births in high‐income countries with midwifery education in accordance with ICM criteria. Case series and case reports were also considered for maternal and neonatal mortality and hospital transfer. Studies had to be published in English, Swedish, Norwegian or Danish. Expected low‐risk births were defined as singletons, cephalic position, spontaneous onset of labour, normal blood pressure, no previous caesarean section and full term at start of labour (w 37 + 0–41 + 6) [14] allowing studies to include up to 5% post‐term births (≥ 42 + 0) as labour starting at 41 + 6 gestational weeks will, in some cases, lead to birth after 42 + 0 gestational weeks. Birth settings were defined according to the National Institute for Health Research Service Delivery and Organization program [15]. Studies were required to report at least one of the defined outcomes to be included. Because both the pregnant population and obstetric care have changed substantially over the past 20 years in all high‐income settings—for example, increasing maternal age and BMI as well as rising rates of labour induction and caesarean section—only studies published after 2004 were included.

**FIGURE 1:**
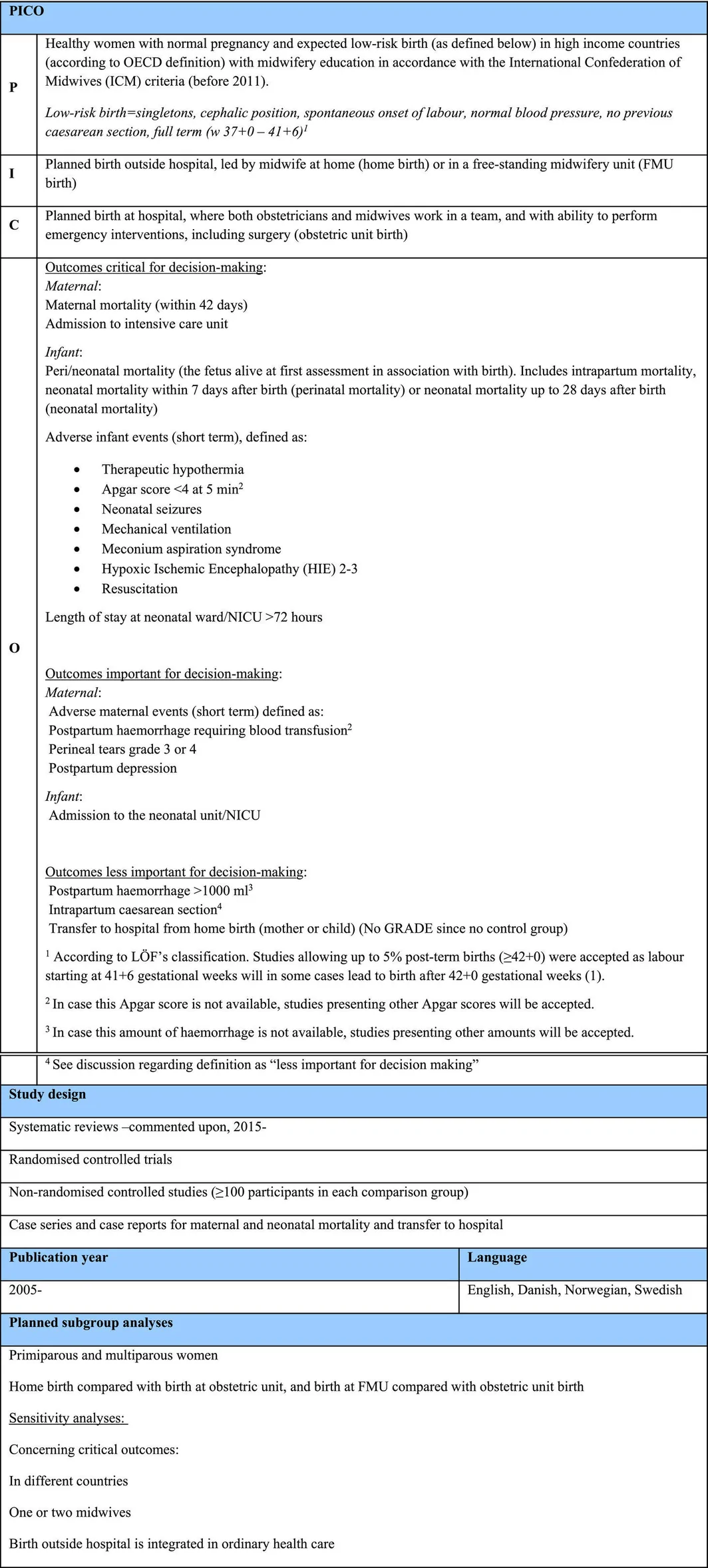
PICO framework defining the population, intervention, comparator and outcomes used to determine study eligibility for this systematic review.

### 2.3 Search Strategy and Study Selection

A comprehensive literature search was conducted by two experienced medical librarians in systematic literature searches, in May 2024, March 2025, and updated on April 8, 2026. Two librarians performing the literature search aligns with international best‐practice guidelines and improves search quality. Peer review reduces errors and bias. Databases searched included Medline, Embase, the Cochrane Library and CINAHL. In addition, the websites of Scandinavian national and regional HTA organisations were screened, and the reference lists of relevant reports were manually reviewed. The two authors independently screened all titles and abstracts using Rayyan software [16]. Potentially eligible studies were retrieved in full text and assessed for inclusion independently by at least two authors; disagreements were resolved in consensus meetings with all authors (PRISMA flow diagram, Figure 2). Detailed search strategies are provided in Appendix S1.

**FIGURE 2:**
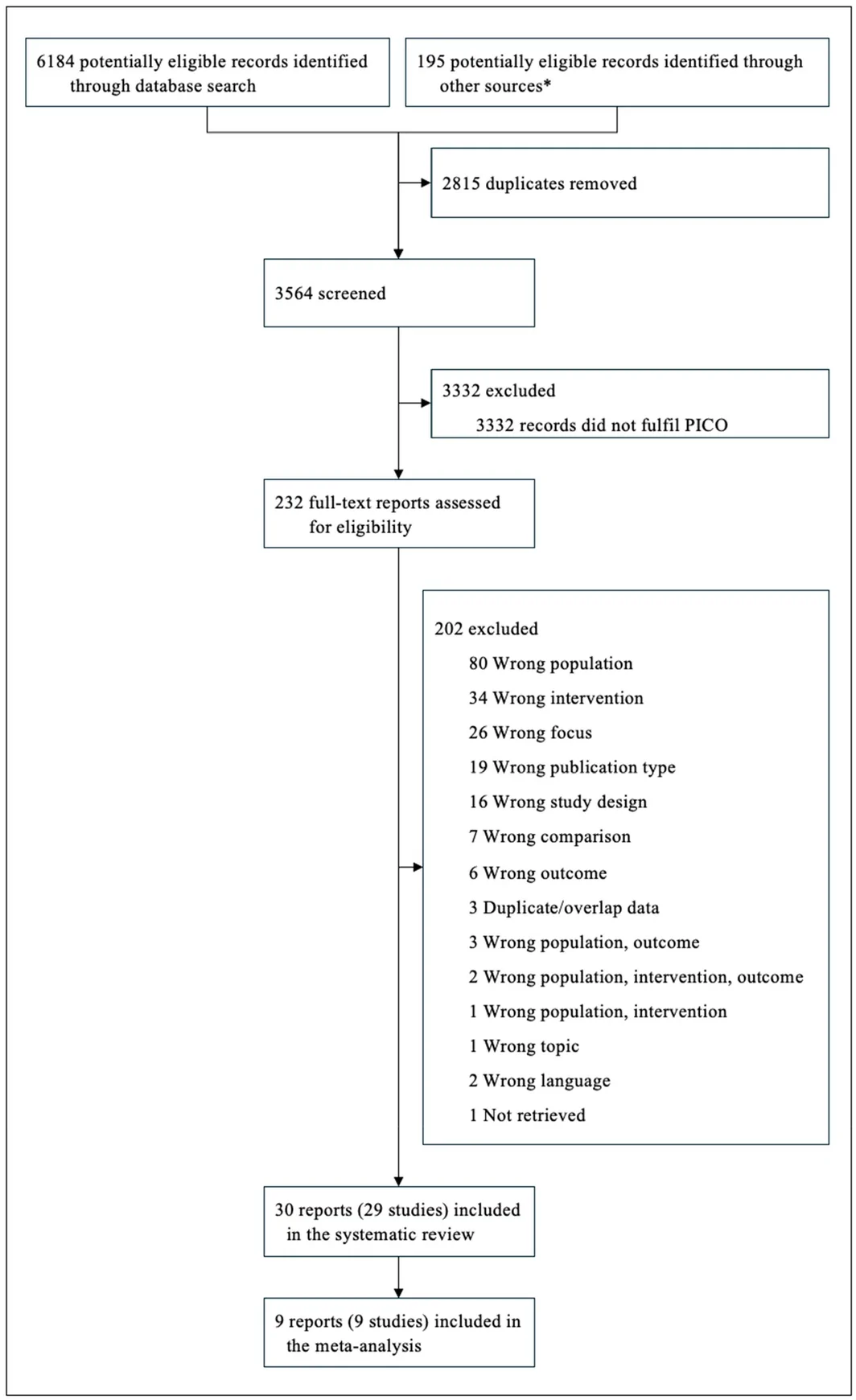
PRISMA 2020 flow diagram of study selection. The figure summarises the identification, screening, eligibility assessment and inclusion of studies in the systematic review. *Other sources: Webpages and reference lists.

A search of *ClinicalTrials.gov* and the WHO *ICTRP* (November 22, 2024) identified 28 ongoing or recently completed trials on planned home or FMU births, mainly focusing on maternal or neonatal safety outcomes, none of which fulfilled the current PICO. An updated search on May 18, 2026, identified five trials, none fulfilling the PICO.

### 2.4 Data Extraction and Quality Assessment

Data extraction was performed independently by at least two authors. Extracted variables included study design, setting, population characteristics (including parity), planned birth setting, outcomes and effect estimates. Risk of bias in non‐randomised studies was assessed using domains based on the Risk of Bias in Non‐randomised Studies of Interventions (ROBINS‐I) tool, as operationalised by the Swedish agency for health and technology assessment and assessment of social services (SBU). ROBINS‐I is the standard tool used for critical appraisal of non‐randomised trials at the HTA unit and is also used at other HTA units in Sweden such as SBU. Case reports and case series were not assessed using formal risk of bias assessment tools. Certainty of evidence was assessed using the Grading of Recommendations, Assessment, Development and Evaluation (GRADE) approach [17]. Disagreements were resolved through discussion until a consensus was reached.

### 2.5 Patient and Public Involvement

The research team involved multiple professions, including librarians, statisticians, health economists, obstetricians, neonatologists and midwives with experience in both home and obstetric unit births. Women representatives, including both women giving birth at home and at a hospital, contributed to defining the PICO and in reviewing the summary; their feedback was incorporated into the review.

### 2.6 Statistical Analysis

Only cohort studies were included in the meta‐analyses. All outcomes were analysed based on the intended place of birth; for example, caesarean sections following transfer from home were included in the home‐birth group. When possible, data were pooled using random‐effects meta‐analyses (STATA v18), with odds ratios (ORs) and 95% confidence intervals (CIs) as the principal summary measures. The random effects model was chosen as all studies were observational with some differences between the studies regarding the population's characteristics and treatments that may affect the results. Adjusted odds ratios (AORs) were used as the primary effect measure when available. If only one study reported an outcome with AOR, the certainty of evidence was based on ORs. Furthermore, absolute effects, calculated as the total number of events divided by the total number of all included studies, are presented. Thus, the absolute effect may differ from the weighted meta‐analysis results and might even point in the opposite direction.

When studies reported adjusted estimates with 99% confidence intervals, these were recalculated to 95% confidence intervals for meta‐analysis. For outcomes with zero events in one study arm, a continuity correction of 0.5 was applied.

Statistical heterogeneity was assessed using *I* ^2^ statistics. In meta‐analyses including fewer than five studies, heterogeneity was interpreted by visual evaluation of forest plots due to substantial uncertainty in *I* ^2^ estimates.

Subgroup analyses were planned according to parity (primiparous and multiparous women) and planned place of birth (home and FMU birth), along with sensitivity analyses restricted to home/FMU births integrated within the routine healthcare system, according to country and number of assisting midwives at birth.

## 3 Results

### 3.1 Study Selection and Characteristics

A total of 6184 and 195 records were identified through databases or other sources, respectively. After deduplication, 3564 were screened by title and abstract. No systematic reviews or randomised controlled trials met the PICO. The literature search identified nine cohort studies [3, 4, 18, 19, 20, 21, 22, 23, 24] (*n* = 1 261 312), 18 case series [25, 26, 27, 28, 29, 30, 31, 32, 33, 34, 35, 36, 37, 38, 39, 40, 41, 42], three case reports [43, 44, 45] and five cost analyses [36, 46, 47, 48, 49] that fulfilled the inclusion criteria (see Figure 2). Of the nine cohort studies, eight [3, 4, 18, 19, 20, 21, 22, 23] contributed to the main comparative meta‐analyses of maternal and perinatal/neonatal outcomes. The remaining cohort study by Scarf et al. [24] was included only for the outcome transfer to hospital and in subgroup analyses since otherwise overlapping with the study by Homer et al. [4]. The characteristics of the included studies are summarised in Table S1a, and the excluded studies with reasons for exclusion are listed in Table S1b.

Included studies varied in setting, planned place of birth, and population characteristics. Most were performed in countries with integration of out‐of‐hospital births in public healthcare, such as Australia, the Netherlands, New Zealand and the United Kingdom. In contrast, Lindgren et al. represents the non‐integrated Swedish setting with privately arranged home births [23]. Seven cohort studies included planned home births only, one study included both home and FMU births [3], and one study included FMU births only [20]. Two cohort studies [20, 22] had serious problems with directness (e.g., setting, planned place of birth, population characteristics) and four studies [4, 18, 19, 21] had some problems with directness. Three studies [18, 19, 22] had a major risk of bias, and five studies [3, 4, 20, 21, 23] had some problems with risk of bias. Depending on specific outcomes, one study [18] had no problems with precision for any outcome, two studies [3, 4] had no or some problems and two studies [22, 23] had serious problems with precision for all outcomes. In two studies [19, 20] the precision varied from no to major problems, and in one study, precision varied from some to major [19] across the reported outcomes. Certainty of evidence was very low for maternal mortality and low for all other outcomes. For the critical appraisal of each study and outcome, see Tables S2 and S3, respectively. For some outcomes, *I* ^2^ values were high, often around 70%–95% (see Figures S1a–S63b). However, we did not downgrade for heterogeneity since there were too few studies to formally evaluate heterogeneity [50].

A summary of the outcomes is presented in Figure 3 (summary forest plots) and Table 1 (summary of findings). Forest plots for the main analyses are presented in Figures S1a–S9b, and weighted risk differences are shown in Figures S10–S18. Assessment across GRADE domains is provided in Table S3.

**FIGURE 3:**
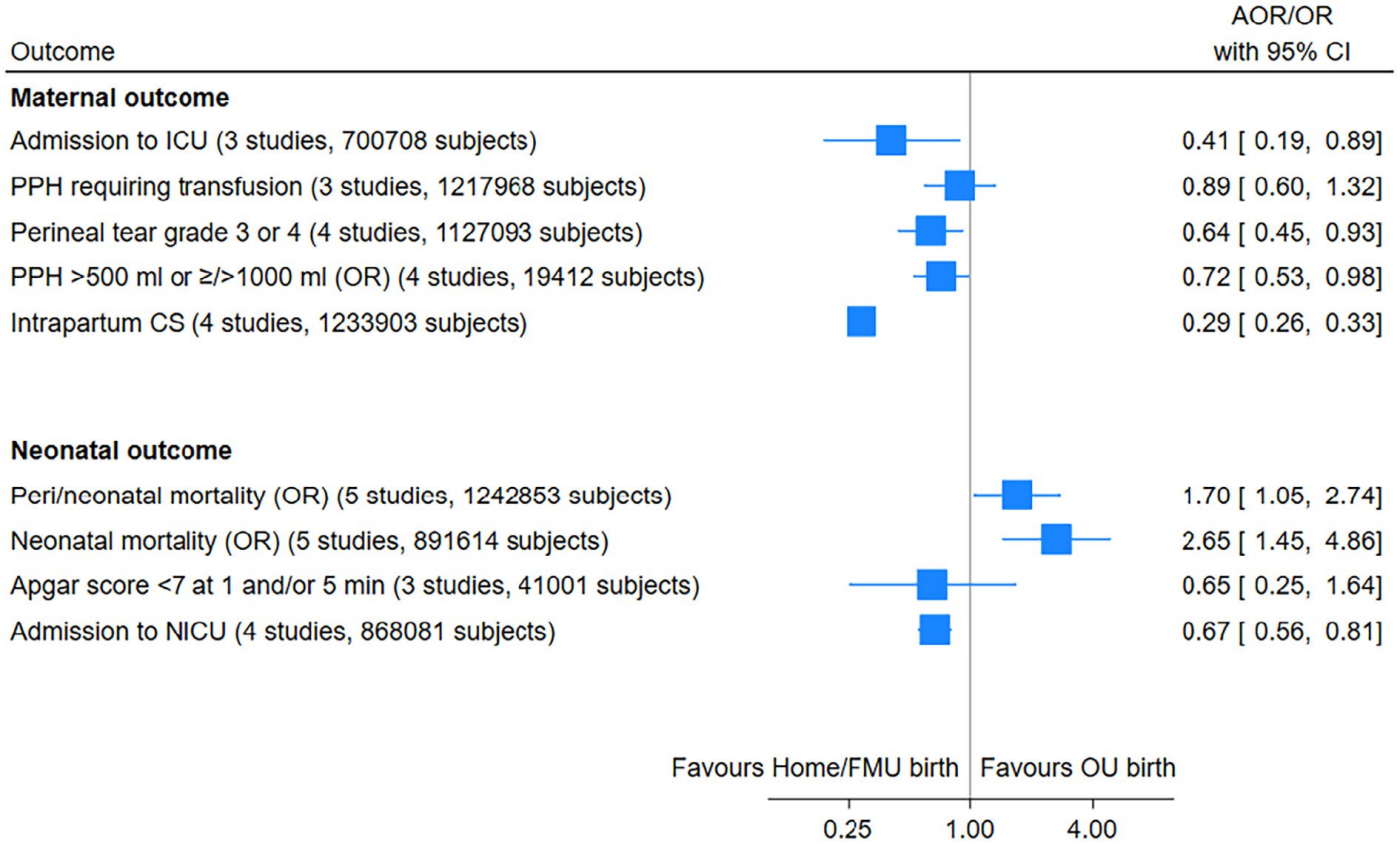
Summary Forest Plot for maternal and neonatal outcomes in planned home/freestanding midwifery unit births versus planned obstetric unit births. AOR, adjusted odds ratio; CS, caesarean section; ICU, intensive care unit; NICU, neonatal intensive care unit; OR, odds ratio; OU, obstetric unit; PPH, postpartum heamorrhage.

**TABLE 1:** Summary of findings of maternal and neonatal outcomes in planned home/freestanding midwifery unit births versus planned obstetric unit births.

Outcome	Number of studies per study design Number of participants	Absolute effect		Relative effect OR/AOR (95% CI)^a^ *p*	Certainty of evidence^b^ GRADE
		HB/FMU births % (*n*/*N*)	OU births % (*n*/*N*)		
Outcomes critical for decision making					
Maternal mortality	3 cohort studies *N* = 18 125 3 case series *N* = 3929	0 (0/2990)	0 (0/15 135)	NA	Very low ⊕ΟΟΟ
Maternal admission to intensive care unit	3 cohort studies *N* = 700 708	0.32 (74/22 949)	0.41 (2764/677 759)	AOR 0.41 (0.19 to 0.89) *p* = 0.02	Low ⊕⊕ΟΟ
Peri‐/neonatal mortality^c^ (intrapartum intrauterine foetal death and neonatal mortality within 7 or 28 days)	5 cohort studies *N* = 1 242 853 8 case series *N* = 473 233 2 case reports	0.08 (24/28 753) In case series for home births between 0 to 0.52%	0.07 (899/1 214 100)	OR 1.70 (1.05 to 2.74) *p* = 0.03	Low ⊕⊕ΟΟ
Neonatal mortality^c^ (within 7 or 28 days)	5 cohort studies *N* = 891 614 7 case series *N* = 472 738 2 case reports	0.05 (14/26278) In case series for home births between 0 to 0.52%	0.03 (236/891 614)	OR 2.65 (1.45 to 4.86) *p* < 0.01	Low ⊕⊕ΟΟ
Apgar score < 7 at 1 and/or 5 min^d^	3 cohort studies *N* = 41 001	0.8 (147/18082)	1.0 (226/22919)	AOR 0.65 (0.25 to 1.64) *p* = 0.36	Low ⊕⊕ΟΟ
Outcomes important for decision making					
Postpartum haemorrhage requiring transfusion	3 cohort studies *N* = 1 217 968	0.6 (159/25985)	0.5 (6482/1 191 983)	AOR 0.89 (0.60 to 1.32) *p* = 0.56	Low ⊕⊕ΟΟ
Perineal tear grade 3 or 4	4 cohort studies *N* = 1 127 093	1.6 (418/26072)	2.0 (22 091/1101029)	AOR 0.64 (0.45 to 0.93) *p* = 0.02	Low ⊕⊕ΟΟ
Admission to neonatal ward or NICU	4 cohort studies *N* = 868 081	1.4 (344/24 685)	0.9 (7603/843 396)	AOR 0.67 (0.56 to 0.81) *p* < 0.01	Low ⊕⊕ΟΟ
Outcomes less important for decision making					
Postpartum haemorrhage (> 500 mL or ≥/> 1000 mL)	4 cohort studies *N* = 18 668	4.1 (163/4176)	4.0 (581/15 236)	OR 0.72 (0.53 to 0.98) *p* = 0.04	Low ⊕⊕ΟΟ
Intrapartum caesarean section	4 cohort studies *N* = 1 233 903	2.6 (719/27975)	7.9 (95 580/1 205 928)	AOR 0.29 (0.26 to 0.33) *p* < 0.01	Low ⊕⊕ΟΟ

Abbreviations: AOR, adjusted odds ratio; FMU, freestanding midwifery unit; HB, home birth; NA, not applicable; NICU, neonatal intensive care unit; OR, odds ratio; OU, obstetric unit.

^a^ Effect estimates are presented as odds ratios (ORs) with 95% confidence intervals (CIs). Adjusted odds ratios (AORs) were used when available; otherwise, unadjusted ORs were calculated from reported data.

^b^ Certainty of evidence was assessed using the Grading of Recommendations, Assessment, Development and Evaluation (GRADE) approach for observational studies. GRADE Working Group grades of evidence: High certainty—very confident that the true effect lies close to the estimate. Moderate certainty—the true effect is likely close to the estimate but may be substantially different. Low certainty—the true effect may be substantially different from the estimate. Very low certainty—the true effect is likely to be substantially different from the estimate. Conclusions were based on AOR when adjusted data were pooled from *more than one* study. If AOR was based on only one study, conclusions were based on OR (with several studies). Downgrading decisions were based on risk of bias, indirectness, imprecision and inconsistency.

^c^ Perinatal mortality includes intrapartum deaths and neonatal deaths as defined in the original studies.

^d^ Apgar score < 7 at 1 and/or 5 min was used as prespecified Apgar score < 4 at 5 min was not available.

### 3.2 Outcomes Critical for Decision Making

Maternal mortality was reported in three cohort studies [20, 21, 23] (including 2990 home/FMU births and 15 135 obstetric unit births) and in three case series [29, 32, 41] (including 3929 planned home/FMU births). No maternal deaths were reported across all studies (22 054 participants in total) (very low certainty of evidence).

Maternal admission to intensive care unit (ICU) was reported in three cohort studies [3, 4, 20] including 22 949 planned home/FMU births and 677 759 obstetric unit births. The crude event rate was 0.3% in the home/FMU group compared with 0.4% in the obstetric unit group. Meta‐analysis showed a lower risk of ICU admission after planned home/FMU birth (OR 0.36, 95% CI 0.19–0.70), which was confirmed in adjusted analyses (AOR 0.41, 95% CI 0.19–0.89) (low certainty of evidence).

Peri/neonatal mortality (defined as perinatal mortality including intrapartum mortality and neonatal mortality within 7 days after birth and neonatal mortality including death of a lifeborn infant up to 28 days after birth) was reported in five cohort studies [3, 4, 19, 20, 23] including 28 753 planned home/FMU births and 1 214 155 obstetric unit births, as well as eight case series [29, 30, 31, 32, 35, 40, 41, 42] (*n* = 473 233) and two case reports [43, 44]. Across cohort studies, crude event rates were 0.8 per 1000 births in the home/FMU group compared with 0.7 per 1000 in obstetric unit births. Pooled unadjusted analysis showed an increased risk of peri/neonatal mortality in planned home/FMU births compared with obstetric unit births (OR 1.70, 95% CI 1.05–2.74) (low certainty of evidence).

Neonatal mortality within 28 days was reported in five cohort studies [3, 4, 19, 20, 23] (26 278 planned home/FMU births, 865 391 obstetric unit births), seven case series [29, 30, 31, 32, 40, 41, 42], and three case reports [43, 44, 45]. Pooled unadjusted analysis of cohort studies showed a higher risk of neonatal death in the home/FMU group (OR 2.65, 95% CI 1.45–4.86), with crude event rates of 0.5 per 1000 versus 0.3 per 1000 (low certainty of evidence). Among seven case series (*n* = 472 738), neonatal mortality rates ranged from 0 to 5.2 per 1000 births, with 0.4 per 1000 in the largest study [31].

Further specified neonatal outcomes, including therapeutic hypothermia, Apgar score < 4 at 5 min, neonatal seizures, mechanical ventilation, meconium aspiration syndrome, hypoxic–ischemic encephalopathy (HIE) grade 2–3, neonatal resuscitation, and admission to neonatal ward or neonatal intensive care unit (NICU) stay > 72 h were not reported in any of the included studies.

Apgar score < 7 at 1 and/or 5 min were reported in three cohort studies [3, 20, 22] including 18 082 neonates in the planned home/FMU group and 22 919 neonates in the obstetric unit group. The crude event rates were 0.8% in the home/FMU group and 1.0% in the obstetric unit group. Meta‐analysis showed no significant difference between the groups (OR 0.68, 95% CI 0.30–1.53; AOR 0.65, 95% CI 0.25–1.64) (low certainty of evidence).

### 3.3 Outcomes Important for Decision‐Making

Postpartum haemorrhage requiring transfusion was reported in three cohort studies [3, 4, 21] including 25 985 planned home/FMU births and 1 191 983 obstetric unit births. The crude event rate was 0.6% versus 0.5%, and meta‐analysis showed no significant difference (OR 0.72, 95% CI 0.44–1.19; AOR 0.89, 95% CI 0.60–1.32) (low certainty of evidence).

Perineal tears grade III–IV were reported in four cohort studies [3, 4, 21, 22] including 26 072 planned home/FMU births and 1 101 021 obstetric unit births. Crude event rates were 1.6% versus 2.0%, and while unadjusted analyses showed no difference (OR 0.72, 95% CI 0.35–1.48), adjusted analyses indicated a reduction in severe tears after home/FMU birth (AOR 0.64, 95% CI 0.45–0.93) (low certainty of evidence).

The outcome postpartum depression was not reported in any of the included studies.

Any admission to a neonatal ward or NICU was reported in four cohort studies [3, 4, 20, 21] with 24 685 neonates in the home/FMU group and 843 396 in the obstetric unit group. Crude event rates were 1.4% in the home/FMU group and 0.9% in the obstetric unit group. Meta‐analysis showed a lower rate of NICU admission after planned home/FMU birth compared with obstetric unit birth (OR 0.60, 95% CI 0.53–0.68; AOR 0.67, 95% CI 0.56–0.81) (low certainty of evidence).

### 3.4 Outcomes Less Important for Decision‐Making

Postpartum haemorrhage (> 500 or ≥/> 1000 mL) was reported in four cohort studies [19, 20, 21, 22] including 4176 planned home/FMU births and 15 236 obstetric unit births. Crude event rates were 4.1% versus 4.0%. Meta‐analysis showed reduced risk of postpartum haemorrhage in planned home/FMU births (OR 0.72, 95% CI 0.53–0.98; AOR 0.54, 95% CI 0.42–0.68) (low certainty of evidence).

Intrapartum caesarean section was reported in four cohort studies [3, 4, 19, 20] including 27 975 planned home/FMU births and 1 205 928 obstetric unit births, and the crude event rates were 2.6% versus 7.9%, respectively. Meta‐analysis indicated a lower risk of intrapartum caesarean section after planned home/FMU birth (OR 0.24, 95% CI 0.21–0.28; AOR 0.29, 95% CI 0.26–0.33) (low certainty of evidence).

### 3.5 Transfer to Hospital

Transfer rates to hospitals during labour or after birth varied across studies and were reported in four cohort studies [20, 21, 22, 24] and 16 case series [25, 26, 27, 28, 29, 32, 33, 34, 35, 37, 38, 39, 40, 41, 42, 51] (some studies included overlapping populations). Four case series only provided data for nulliparous women, because data for multiparous women were either not reported separately or did not meet the low‐risk criteria (e.g., women with previous caesarean section were included). Therefore, these studies were included only in the nulliparous subgroup analyses [27, 34, 35, 39]. In the overall transfer summary, four cohort studies [20, 21, 22, 24] (*n* = 4192) and the remaining 12 case series [25, 26, 28, 29, 32, 33, 37, 38, 40, 41, 42, 51]; (*n* = 24 538) reported transfer rates ranging from 9.3% to 33.3%, with urgent transfer occurring in 3.4% of cases.

### 3.6 Subgroup Analyses According to Parity

Maternal and neonatal outcomes according to parity are presented in Figures S19a–S27b for nulliparous women and Figures S28a–S36b for multiparous women.

Among nulliparous women, meta‐analysis of four cohort studies [3, 4, 21, 24] (*n* = 210 090) and two case series [31, 32] (*n* = 198 811) showed a higher risk of peri/neonatal mortality for planned home/FMU births compared with obstetric unit births (six studies; *n* = 408 901; OR 2.12, 95% CI 1.02–4.44), with transfer rates ranging from 14.4% to 60.6%, and urgent transfers from 4.5% to 25.8% (one cohort study [20] and 11 case series [27, 28, 32, 33, 34, 35, 37, 39, 40, 42, 51]; *n* = 267 316).

Among multiparous women, meta‐analysis of four cohort studies [3, 4, 21, 24] (*n* = 961 399) and two case series [31, 32] (*n* = 268 050) showed no difference in peri/neonatal mortality (six studies, *n* = 1 229 449; OR 1.18, 95% CI 0.57–2.47). Reported transfer rates among multiparous women were substantially lower than those among nulliparous women, ranging from 2.8% to 23% during labour and from 2.4% to 2.8% after birth (one cohort study [20] and eight case series [28, 32, 33, 37, 39, 40, 42, 51]; *n* = 272 772).

### 3.7 Subgroup Analyses According to Planned Place of Birth

Maternal and neonatal outcomes according to the planned place of birth subgroup analysis are presented in Figures S37a–S45b for home births and Figures S46a–S54b for FMU births.

Meta‐analyses comparing home births versus obstetric unit births showed a higher peri/neonatal mortality (five cohort studies [3, 4, 19, 21, 23]; *n* = 28 753; OR 1.70, 95% CI 1.05–2.74) and neonatal mortality (four cohort studies [3, 4, 19, 23]; *n* = 26 278; OR 2.71, 95% CI 1.46–5.01). Transfer rates ranged between 10.7% and 33.3%, with urgent transfers reported in 3.4%–4.9% of cases (three cohort studies [3, 21, 22] and 12 case series [25, 26, 28, 29, 32, 33, 37, 38, 40, 41, 42, 51]; *n* = 27 596).

For FMU births versus obstetric unit births, no significant differences were observed in peri/neonatal mortality (two cohort studies [3, 20]; *n* = 35 195). Transfer rates ranged from 21.7% to 21.9%, and potentially urgent transfers occurred in 6.5% of cases (one cohort study [20] and one case series [51]; *n* = 12 311).

### 3.8 Sensitivity Analyses

Sensitivity analyses for home/FMU births integrated into the ordinary healthcare system did not indicate differences in peri/neonatal mortality (two cohort studies [3, 21]; *n* = 38 332; OR 1.08, 95% CI 0.22–5.20) (Figures S55–S63b).

Planned analyses based on country or number of assisting midwives were not feasible due to insufficient data.

### 3.9 Economic Consequences

Five studies met the inclusion criteria [36, 46, 47, 48, 49] (Table S4). The cost analysis studies were context dependent and differed regarding their analytical perspective (e.g., healthcare or societal), the costing methods used—such as the top‐down approach, which allocates total health service expenditures to individual services using aggregate data, or the bottom‐up (micro‐costing) approach, which identifies, measures, and values each individual resource utilised [52] as well as used data sources (primary data collection versus secondary databases) and types of cost components included. Five economic evaluations from Australia, England, Norway, and Italy reported lower direct costs for planned home/FMU births than for hospital births, with reductions ranging from 10% to 61%. One study [48] reported slightly higher costs for home births (2.5% higher than hospital births). However, when the costs of on‐call midwifery services and emergency standby capacity were included, the cost advantage diminished or reversed [36].

## 4 Discussion

This is the first systematic review and meta‐analysis to evaluate maternal and peri/neonatal mortality and severe morbidity in planned midwife‐assisted home/FMU births compared with obstetric team assisted hospital births among women with expected and strictly defined low‐risk births in high‐income settings. Midwifery education had to be specified as being in accordance with the ICM criteria [9] and AMU births were not included. We identified no randomised controlled trials and included nine cohort studies, 18 case series, and three case reports. The certainty of evidence was low for all outcomes, except for maternal mortality, where results had very low certainty of evidence.

No maternal deaths were reported in the included studies, that were underpowered to detect such rare events. Home/FMU birth may be associated with outcomes critical for decision making, for example, reduced maternal admission to intensive care, and increased peri/neonatal mortality—especially among nulliparous women. No difference was found in low Apgar scores at 1 or 5 min. Further neonatal outcomes specified as critical for decision making—such as HIE (grade II‐III), mechanical ventilation, seizures, or therapeutic hypothermia—were not reported, highlighting important gaps in the evidence. Further, home/FMU births may be associated with no difference in postpartum haemorrhage requiring transfusion, a lower rate of third‐ and fourth‐ degree perineal tears, and reduced admission to NICU compared with obstetric unit birth. Home/FMU births may also be associated with reduced risk for postpartum haemorrhage and intrapartum caesarean section. Transfer rates from home/FMU births to hospital care occurred in a notable proportion of cases. No study reported data on postpartum depression.

This review has several strengths, including a clearly defined PICO framework, systematic grading of outcomes, and involvement of multiple professional perspectives, including staff assisting in both home and obstetric unit births and women representatives. Limitations include reliance on observational studies only, absence of hard outcome measures in many studies, risk of selection bias as more healthy women have chosen home/FMU births, and insufficient sample sizes to detect rare events. Moreover, most data originated from high‐income countries with well‐integrated out‐of‐hospital birth settings within the healthcare systems, limiting generalisability to less integrated settings.

Health economic analyses were limited by uncertainty regarding severe medical outcomes and the lack of costs for emergency stand‐by capacities for home/FMU births.

The findings must be interpreted considering methodological limitations. All the included studies were observational, introducing potential bias. Women in the home/FMU group were more often multiparous, native speakers, had higher education and socioeconomic status, and had fewer medical risk factors, suggesting a risk for selection bias and residual confounding. The higher proportion of multiparous women in the home/FMU group is particularly relevant, as the increased peri/neonatal mortality in home/FMU births observed in this review was confined mainly to nulliparous women. Outcomes based on subjective clinical assessment—such as postpartum haemorrhage, perineal tears or Apgar scores—may be diagnosed or reported differently due to differences in documentation and surveillance, others may be influenced by availability, for example, neonatal intensive care, while more robust endpoints such as perinatal or neonatal mortality are less susceptible to such bias. Such reporting bias is suggested by the findings of lower postpartum haemorrhage rates in home/FMU births without a corresponding reduction in transfusion. Similarly, while no difference was found in low Apgar scores, neonatal mortality was consistently higher in planned home/FMU births than in obstetric unit births. It should be noted that Apgar scores have a low predictive value for adverse child outcomes [53]; however, as they are included in almost all studies on the topic, we chose to include this measure to increase comparability with previous research. Further, reduced caesarean section rates and NICU admissions must be interpreted in the context of increased peri/neonatal mortality in planned home/FMU births.

Our findings are partly consistent with previous systematic reviews. Hutton et al. [7] and Scarf et al. [6] reported no significant difference in perinatal or neonatal mortality in well‐integrated healthcare systems, though their populations included mixed‐risk births and heterogenous hospital comparison groups including AMUs. In contrast, this review focused exclusively on expected and strictly defined low‐risk births and included both integrated and less‐integrated healthcare systems, finding a consistent increase in peri/neonatal mortality for planned home/FMU births. Maternal outcomes are supported by previous evidence, showing fewer interventions, lower rates of severe perineal trauma in home/FMU births, and no association with postpartum haemorrhage or ICU admission [6, 7]. It can be assumed that the differences between study populations increase when studies include births with intermediate risk, thereby further raising the likelihood of residual confounding and selection bias, particularly for outcomes that may be distorted in the opposite direction of the bias. To minimise these risks, this study focused exclusively on strictly defined low‐risk births. The staffing and competence of staff assisting the birth can be seen as part of the birth setting; therefore, this metanalysis defined midwife competence and restricted the control group to obstetric unit births where obstetric competence is part of the team.

Ethical considerations must take into account respect for women's autonomy and informed decision‐making, as well as medical safety for both the mother and child. In addition, allocating resources to healthy women with expected low‐risk births may divert capacity from patients with greater medical needs. Given the low certainty of evidence, future research should prioritise well‐designed prospective observational studies with clearly defined low‐risk criteria, comparison setting, robust adjustments for confounders, and comprehensive reporting of both maternal and peri/neonatal outcomes. Large registry‐based studies may provide valuable insights, particularly in healthcare systems where home/FMU births are less integrated.

## 5 Conclusion

In conclusion, scientific evidence regarding maternal and neonatal safety in planned, expected low‐risk home/FMU births in high‐income settings with midwifery education in accordance with the ICM criteria [9] remains low to very low. Home/FMU births may be associated with an increase in peri/neonatal mortality, but fewer maternal complications compared to obstetric unit births. These findings highlight the need for careful counselling, organising access to hospital care, and further high‐quality research to strengthen the evidence base.

## Author Contributions

**Anders Elfvin:** conceptualization, writing – review and editing, funding acquisition, investigation. **Sara Holm:** conceptualization, writing – review and editing, investigation. **Carolina Herding:** conceptualization, writing – original draft, investigation, visualization. **Karolina Linden:** conceptualization, writing – review and editing, funding acquisition, investigation. **Ulla‐Britt Wennerholm:** conceptualization, writing – review and editing, methodology, investigation, visualization. **Ylva Carlsson:** conceptualization, writing – review and editing, project administration, investigation. **Ida Stadig:** conceptualization, methodology. **Petteri Sjögren:** conceptualization, writing – review and editing, investigation. **Ann Liljegren:** conceptualization, methodology, writing – review and editing. **Christina Bergh:** conceptualization, writing – review and editing, investigation. **Verena Sengpiel:** conceptualization, writing – original draft, supervision, project administration, funding acquisition, investigation. **Max Petzold:** conceptualization, formal analysis, writing – review and editing, visualization. **Jahangir Khan:** conceptualization, writing – review and editing, investigation.

## Funding

This study was conducted at the Centre for Health Technology Assessment (HTA), Region Västra Götaland, assessing HTA report applications for priority. A.E., V.S. and K.L. have been supported by the Swedish state under the agreement between the Swedish government and the county councils, the ALF‐agreement (AE ALFGBG‐1006755, KL ALF‐GBG‐1006131, and VS ALFGBG‐970689).

## Conflicts of Interest

The authors declare no conflicts of interest.

## Supporting information


**Table S1a:** Characteristics of included studies.
**Table S1b:** Excluded studies with reason for exclusion.
**Table S2:** Risk of bias (ROBINS‐I), study level.
**Table S3:** Certainty of evidence (GRADE), outcome level.
**Table S4:** Economic evaluations.
**Appendix S1:** Search strategy.
**Figures S1a–S9b:** Main analysis: Planned home/FMU births vs. planned obstetric unit births.
**Figures S10–S18:** Main analysis: Weighted risk difference (RD) with 95% confidence interval (CI) for planned home/FMU births vs. planned obstetric unit births.
**Figures S19a–S36b:** Subgroup analyses according to parity.
**Figures S37a–S54b:** Subgroup analyses according to place of birth.
**Figures S55‐S63b:** Sensitivity analysis of births outside hospital integrated in ordinary health care vs. obstetric unit births.

## Data Availability

All data relevant to the study are included in the article or as Supporting Information. Additional data are available from the corresponding author upon reasonable request.
