# Supplementary material for: Maternal and Peri/Neonatal Mortality and Morbidity in Midwife‐Assisted Low‐Risk Birth Outside Hospital Compared to Obstetric Team‐Assisted Low‐Risk Hospital Birth: A Systematic Review and Meta‐Analysis
Source: Acta Paediatr. 2026 Jul 20;115(12):2659–70. doi: 10.1111/apa.70685 (PMC13747683; doi:10.1111/apa.70685)
Supplement: Supplementary file 1 [file APA-115-2659-s001.docx]

**Supplementary file**

Table of contents

- Supplementary Table S1a: Characteristics of included studies
- Supplementary Table S1b. Excluded studies with reason for exclusion
- Supplementary Table S2. Risk of bias (ROBINS-I), study level
- Supplementary Table S3. Certainty of evidence (GRADE), outcome level
- Supplementary Appendix S1. Search strategy
- Supplementary Figures S1a-S9b. Main analysis: Planned home/FMU births vs obstetric unit births
- Supplementary Figures S10-18 Main analysis: Weighted risk difference (RD) with 95% confidence interval (CI) for planned home/FMU births vs obstetric unit births
- Supplementary Figures S19a-S36b. Subgroup analyses according to parity
- Supplementary Figures S37a-54b. Subgroup analyses according to place of birth
- Supplementary Figures S55-63b Sensitivity analysis of births outside hospital integrated in ordinary health care vs obstetric unit births
- Supplementary Table S4. Economic evaluations

Supplementary Table S1a: Characteristics of included studies

| **Study, year of publication, country** | **Study design** | **Study period** | **Study groups: Home birth (HB); Freestanding midwifery-led unit (FMU); Obstetric unit (OU)** | **Sample size** | **Percentage nulliparous women** | **Outcomes according to PICO*** | **Comment** |
| --- | --- | --- | --- | --- | --- | --- | --- |
| **Cohort studies** | | | | | | | |
| Brocklehurst (Birthplace in England Collaborative Group), 2011  England | Prospective cohort | 2008-2010 | HB  OU  FMU | HB: 16 840  OU: 1 970  FMU: 11 282 | HB: 27.2%  OU: 54.0%  FMU: 46.0% | 3,4,6,8,9,10,11,12,13 | Postterm ≥42+0 w:  HB: 1.8%  FMU: 1.0%  OU: 2.7%  Partly overlapping with Rowe, 2013 regarding transfer |
| Davis, 2012  New Zealand | Retrospective cohort | 2006-2007 | HB  OU | HB: 1 830  OU: 11 503 | Mean parity (SD):  HB: 1.4 (1.4) OU secondary hospital: 0.9 (1.2)  OU tertiary hospital: 0.7 (1.0) | 13 | Same cohort as Davis, 2011 |
| Davis, 2011  New Zealand | Retrospective cohort study | 2006-2007 | HB  OU | HB: 1 830  OU: 11 503 | Mean parity (SD):  HB 1.4 (1.4) OU secondary hospital 0.9 (1.2) OU tertiary hospital 0.7 (1.0) | 4,8,9,10,11,13 | Only outcome given with absolute numbers is mode of birth, otherwise only: RR with primary unit (FMU+AMU) as reference.  Same cohort as Davis, 2011 |
| Farry, 2019  New Zealand | Retrospective cohort study | NR | OU  FMU (3 different FMUs) | OU: 3 093  FMU: 1 114 | OU: 39.1%  FMU: 32.4% | 1,2,9,10,12,13 | No home deliveries.  No primary outcome. |
| Hermus, 2017  The Netherlands | Prospective  cohort study | 2014 | HB  OU | HB: 1 086  OU: 701 | HB: 36.7%  OU: 49.6% | 1,3,4,6,9,12,13 | 8.5% missing data. |
| Hiraizumi, 2013  Japan | Retrospective cohort study | 2007-2011  (no exact date) | HB  OU | HB: 168  OU: 217 | HB: 24%  OU: 26% | 4,10,12,13 | NB CS, VE table 1 and 2 does not agree, these outcomes cannot be presented, also transfer rates figures in table 2 and 3 do not agree.  Partly overlapping with Suzuki, 2016 |
| Homer, 2019  Australia | Retrospective population  based cohort study | 2000-2012 | HB  OU  FMU not available, presented as “Birth centers” (AMU and FMU) | HB: 8 212  OU: 1 171 703 | HB: 27.9%  OU: 42.2% | 2,3,4,6,7,9,11 | Partly overlapping with Catling –Paull 2013 and Davies Tuck 2018 regarding stillbirth and  Neonatal death.  Transfer rates not available |
| Lindgren, 2008a  Sweden | Retrospective cohort study | 1992-2004 | HB  OU | HB: 897  OU:11 341  Low risk:  HB: 790  OU: 11 195 | HB 25.5%  OU 62% | 1,6 | A randomly selected control group was identified from the Medical Birth Register |
| Scarf, 2019  Australia^#^ | Retrospective population  based cohort study | 2000-2012 | HB  OU  FMU not available, only “Birth centers” (AMU and FMU) | HB: 1 824  OU: 464 630 | HB:42.7%  OU:82.2% | 6,7,9,11,12 | Partly overlap with Homer 2019  *BC are both FMU and AMU, no separate analysis by type of BC  Partly overlap with Caitling-Paul, 2013 regarding transfer |
| **Case series and case reports** | | | | | | | |
| Alcaraz-Vidal, 2024  Spain | Case series | 2016-2017 | HB | HB: 750 | HB 43.6% | 12 |  |
| Amelink-Verburg, 2008  The Netherlands | Retrospective case series | 2001-2003 | HB | HB+AMU  280,097  60.2% intended HM | NR | 12 | *No definition of low risk  Partly overlap with Offerhaus, 2013 |
| Blix, 2016  Norway, Sweden, Denmark, Iceland | Retrospective case series | 2008-2013 (country-specific) | HB | HB: (Norway 482,  Sweden 445,  Denmark 1 843,  Iceland 298) | 18.6% | 12 | Data will be reported only for nulliparous women since 4.7% CS in multiparous women |
| Bolten, 2016  The Netherlands | Retrospective case series | 2009-2011 | HB | HB: 2 050 | 42.3%  57.7% | 12 | Partly overlapping with Geerts 2014 |
| Catling-Paull, 2013  Australia | Retrospective case series | 2005-2010 | HB | HB: 1 807 | 31.8% | 1,6,7,12 | *No definition of low risk, no data on previous CS  Partly overlap with Davies Tuck 2018, McMurtrie 2009, Scarf 2019 |
| Davies Tuck, 2018  Australia | Retrospective case series | 2000-2015 | HB  No OU: OU group also includes BC/AMU births | HB: 3 202 | 34.5% | 6,7 | High risk HB as reported in the article not included in this analysis.    NND not defined as early or late.    Not specified whether stillbirth combines antepartum and intrapartum stillbirth.  Partly overlapping with Catling-Paull 2013, Homer 2019. |
| de Jonge, 2015  The Netherlands | Retrospective case series | 2000-2009 | HB  No OU: OU group also includes BC/AMU births | HB: 466 112 (62.7%) | 42.6% | 6,7 | 8.5% unknown.  Probabilistic data linkage  No OU=therefore included as case series  Partly overlapping with Smit  2014a and b |
| Fritschel, 2015  USA | Case report | 2014 | HB | 1 case |  | 7 | Fatal legionellosis after water birth |
| Galera-Barbero, 2021  Spain | Retrospective case series | 1989-2019 | HB | 820 | 36.1% | 1,6,7,12 | 42+ w: n=3 (<0.5%)  More outcomes in article |
| Geerts, 2014  The Netherlands | Prospective case series | 2009-2010 | HB  No OU: Hospital births were midwifery led | HB:1279 | 41.3% | 12 | Same population included in Bolten 2016 |
| Hill, 2024  Germany | Retrospective case series | 2010-2015 | HB  FMU | HB/FMU: 23 160  Data not presented separately for HB and FMU | 23 160 nulliparous | 12 |  |
| Huitfeldt, 2016  Norway | Retrospective case series | 2007-2011 | FMU | FMU: 495 | 23% | 6,12 | Data will be reported only for nulliparous women since 1.3% CS in multiparous  women |
| Maimburg, 2018  Denmark | Prospective case series | 2015-2017 | HB | HB: 268 | 44% | 12 |  |
| McMurtrie, 2009  Australia | Prospective case series | 2005-2009 | HB | HB:70 | 23% | 12 | Partly overlapping with Catling-Paull 2013 |
| Offerhaus, 2013  The Netherlands | Prospective case series | 2000-2008 | HB | HB: 452 997 | 54.8% | 12 | Partly overlapping with Amelink-Verburg 2008 |
| Rowe, 2013  England | Prospective case series | 2008-2010 | HB  FMU | HB: 16 632  FMU: 11 210 | HB: 27%  FMU:46% | 12 | *Secondary analysis of Brocklehurst 2011 (Birthplace in England Collaborative Group) but >42+0 excluded in Rowe 2013 |
| Smit, 2014a  The Netherlands | Case report  Aims, methods, results in 2014a | 2008-2009 | HB | 8 cases of umbilical cord prolapse | 25% | 6 | Part 1 of article of Smit et al 2014   Probably included into de Jonge 2015 |
| Smit, 2014b  The Netherlands | Case report  Cont. results  in 2014b | 2008-2009 | HB | 8 cases of umbilical cord prolapse | 25% | 6 | Part 2 of article of Smit et al 2014   Probably included into de Jonge 2015 |
| Suzuki, 2016  Japan | Retrospective case series | 2009-2012 | HB | HB: 123 | HB: 26% | 6,12 | Partly overlapping with Hiraizumi 2013 |
| Sweet, 2022  Australia | Retrospective case series | 2009-2019 | HB | HB: 561 | 27.1% | 1,6,8,12 | Partly overlap with Catling-Paull 2013, Davies Tuck 2018, Homer 2019, White 2019 |
| White, 2020  Australia | Case series | 2010-2017 | HB | HB: 191 | 17.3% | 8,12 |  |
| AMU=alongside midwifery unit, BC=birth center, FMU=freestanding midwifery unit; HB=home birth, OU=Obstetric unit  ^#^ The study by Scarf et al., 2019 is only included for the outcome transfer to hospital and in subgroup analyses since otherwise overlapping with the study by Homer et al., 2019.  ***Outcomes (PICO)**  1 Maternal mortality (≤42 days)  2 Admission to intensive care unit  3 Postpartum hemorrhage requiring blood transfusion  4 Severe perineal trauma (grade 3–4)  5 Postpartum depression  6 Perinatal mortality (as defined by the original study)  7 Neonatal mortality (≤7 days or ≤28 days)  8 Severe neonatal morbidity (composite)  9 Admission to neonatal unit or neonatal intensive care unit  10 Apgar score <7 at 5 minutes  11 Intrapartum caesarean section  12 Transfer to hospital from planned home birth (mother or infant)  13 PPH > 500 mL or >1000 mL  Eligibility criteria were similar across studies and generally restricted to planned births among low-risk women with term, singleton, cephalic pregnancies; detailed inclusion and exclusion criteria are reported in the original studies. | | | | | | |  |

Supplementary Table S1b. Excluded studies and reason for exclusion

| **Study** | **Reason for exclusion** |
| --- | --- |
| Alliman J, Phillippi JC. Maternal Outcomes in Birth Centers: An Integrative Review of the Literature. J Midwifery Womens Health. 2016;61(1):21-51. doi: https://dx.doi.org/10.1111/jmwh.12356 | Wrong study design (review, not systematic) |
| Anderson DA, Gilkison GM. The Cost of Home Birth in the United States. Int J Environ Res Public Health. 2021;18(19):01. doi: https://dx.doi.org/10.3390/ijerph181910361. | Cost analysis. Wrong population (not specifically mentioned if the participants are at low-risk concerning mortality and morbidity for mother and child) |
| Andraczek T, Tauscher A, Kabbani N, Todorow H, Stepan H. Comparison of intrapartum transfer from out-of-hospital births with intrapartum transfer from an alongside midwifery unit: a real-world data analysis of a German cohort. J Perinat Med. 2025;53(8):1077-88. doi: https://dx.doi.org/10.1515/jpm-2025-0141. | Wrong study design – comparison transferred out-of-hospitals births to transferred AMU births |
| Bachilova S, Czuzoj-Shulman N, Abenhaim HA. Effect of Maternal and Pregnancy Risk Factors on Early Neonatal Death in Planned Home Births Delivering at Home. J Obstet Gynaecol Can. 2018;40(5):540-6. doi: https://dx.doi.org/10.1016/j.jogc.2017.07.029. | Wrong population (not only women with normal pregnancies and expected normal births) |
| Baczek G, Tataj-Puzyna U, Sys D, Baranowska B. Freestanding Midwife-Led Units: A Narrative Review. Iran J Nurs Midwifery Res. 2020;25(3):181-8. doi: https://dx.doi.org/10.4103/ijnmr.IJNMR_209_19. | Wrong study design (review, not systematic) |
| Baker K, Stephenson J. Third stage of labour management approaches and postpartum haemorrhage in midwife-led units. Br J Midwifery. 2022;30(5):250-6. doi: 10.12968/bjom.2022.30.5.250. | Wrong population, intervention and outcome (case series, not mortality, not transfer, only vaginal birth included) |
| Bayoglu Tekin Y, Mete Ural U, Kerimoglu OS, Balik G, Kir Sahin F, Guvendag Guven ES. Is induction of labour by Propess( R) safe? A comparison of midwife-led versus obstetrician-led labour management. J Obstet Gynaecol. 2015;35(4):346-9. doi: https://dx.doi.org/10.3109/01443615.2014.960829. | Wrong intervention (in hospital) |
| Bellini T, Vinci F, Polleri G, Pezzotta F, Grasselli L, Piccotti E, et al. Adverse Outcomes in Neonates Following Planned Home Births: A Case Report Series and a Narrative Literature Review. J Clin Med. 2025;14(4):11. doi: doi:https://doi.org/10.3390/jcm14041181. | Wrong outcome (case series, not mortality or transfer) |
| Benyshek DC, Cheyney M, Brown J, Bovbjerg ML. Placentophagy among women planning community births in the United States: Frequency, rationale, and associated neonatal outcomes. Birth. 2018;45(4):459-68. doi: https://dx.doi.org/10.1111/birt.12354. | Wrong focus (placentophagy) |
| Bland M. The influence of birth experience on postpartum depression. Midwifery Today Int Midwife. 2009(89):20-66. | Wrong population, intervention and outcome (case series, not mortality, not transfer) |
| Blix E, Huitfeldt AS, Oian P, Straume B, Kumle M. Outcomes of planned home births and planned hospital births in low-risk women in Norway between 1990 and 2007: a retrospective cohort study. Sex Reprod Healthc. 2012;3(4):147-53. doi: https://dx.doi.org/10.1016/j.srhc.2012.10.001. | Wrong population (not only women with normal pregnancies and expected normal births) |
| Bovbjerg ML, Cheyney M, Everson C. Maternal and Newborn Outcomes Following Waterbirth: The Midwives Alliance of North America Statistics Project, 2004 to 2009 Cohort. J Midwifery Womens Health. 2016;61(1):11-20. doi: 10.1111/jmwh.12394. | Wrong population (not only women with normal pregnancies and expected normal births) |
| Bovbjerg ML, Cheyney M, Hoehn-Velasco L, Jolles D, Brown J, Stapleton J, et al. Planned Home Births in the United States Have Outcomes Comparable to Planned Birth Center Births for Low-Risk Birthing Individuals. Med Care. 2024;62(12):820-9. doi: doi:https://doi.org/10.1097/mlr.0000000000002074. | Wrong population (not only women with normal pregnancies and expected normal births) |
| Buchanan C, Kuo S, Minton L, Lee MJ, Choi SY, Soon R. Neonatal Hypoxic Ischemic Encephalopathy and Planned Home Birth. J Midwifery Womens Health. 2022;67(1):69-74. doi: https://dx.doi.org/10.1111/jmwh.13309. | Wrong outcome (case control study, not mortality or transfer) |
| Burns E, Feeley C, Hall PJ, Vanderlaan J. Systematic review and meta-analysis to examine intrapartum interventions, and maternal and neonatal outcomes following immersion in water during labour and waterbirth. BMJ Open. 2022;12(7):e056517. doi: https://dx.doi.org/10.1136/bmjopen-2021-056517. | Wrong population (not only women with normal pregnancies and expected normal births) |
| Burns EE, Boulton MG, Cluett E, Cornelius VR, Smith LA. Characteristics, interventions, and outcomes of women who used a birthing pool: a prospective observational study. Birth. 2012;39(3):192-202. doi: https://dx.doi.org/10.1111/j.1523-536X.2012.00548.x. | Wrong topic (water birth) |
| Burt M, Tumin D, Bates N, Crockett K, Whiteside JL. Maternal and perinatal outcomes of birth center births compared to planned home births: A nation‐wide, propensity‐matched analysis. J Obstet Gynaecol Res. 2025;51(6):1-8. doi: 10.1111/jog.16329. | Wrong design (births transferred during labour are excluded) |
| Caine D. Homebirth and maternal death. Pract Midwife. 2008;11:30-1. | Wrong publication type (editorial) |
| Campiotti M, Campi R, Zanetti M, Olivieri P, Faggianelli A, Bonati M. Low-Risk Planned Out-of-Hospital Births: Characteristics and Perinatal Outcomes in Different Italian Birth Settings. Int J Environ Res Public Health. 2020;17(8):15. doi: https://dx.doi.org/10.3390/ijerph17082718. | Wrong population (not only women with normal pregnancies and expected normal births) |
| Carlson NS, Neal JL, Tilden EL, Smith DC, Breman RB, Lowe NK, et al. Influence of midwifery presence in United States centers on labor care and outcomes of low-risk parous women: A Consortium on Safe Labor study. Birth. 2019;46(3):487-99. doi: https://dx.doi.org/10.1111/birt.12405. | Wrong intervention (in hospital) |
| Chang JJ, Macones GA. Birth outcomes of planned home births in Missouri: a population-based study. Am J Perinatol. 2011;28(7):529-36. doi: https://dx.doi.org/10.1055/s-0031-1272971. | Wrong population (not only women with normal pregnancies and expected normal births) |
| Chantry AA, Guiguet-Auclair C, Rollet C, Pralus M, Dubel-Jam M, Roelens I, et al. Intrapartum and childbirth care and outcomes in midwife-led birth centres in France: A nationwide descriptive study with an analysis of maternal and neonatal transfers. Women & Birth. 2025;38(3):N.PAG-N.PAG. doi: 10.1016/j.wombi.2025.101908. | Wrong intervention (AMU) |
| Cheng YW, Snowden JM, King TL, Caughey AB. Selected perinatal outcomes associated with planned home births in the United States. Am J Obstet Gynecol. 2013;209(4):325.e1-8. doi: https://dx.doi.org/10.1016/j.ajog.2013.06.022. | Wrong population (not only women with normal pregnancies and expected normal births) |
| Cheyney M, Bovbjerg M, Everson C, Gordon W, Hannibal D, Vedam S. Outcomes of care for 16,924 planned home births in the United States: the Midwives Alliance of North America Statistics Project, 2004 to 2009. J Midwifery Womens Health. 2014;59(1):17-27. doi: https://dx.doi.org/10.1111/jmwh.12172. | Wrong population (not only women with normal pregnancies and expected normal births) |
| Christensen LF, Overgaard C. Are freestanding midwifery units a safe alternative to obstetric units for low-risk, primiparous childbirth? An analysis of effect differences by parity in a matched cohort study. BMC Pregnancy Childbirth. 2017;17(1):14. doi: https://dx.doi.org/10.1186/s12884-016-1208-1. | Wrong intervention (AMU) |
| Cicero RV, Zambri F, Grandolfo M, Varone F, Smith M, Colaceci S. Characteristics of out-of-hospital births and perinatal outcomes: data from the Lazio Region, Italy, cross-sectional study from 2019 to 2021. Ann Ist Super Sanita. 2024;60(2):98-106. doi: doi:https://doi.org/10.4415/ann_24_02_03. | Wrong population (not only women with normal pregnancies and expected normal births) |
| Cox KJ, Bovbjerg ML, Cheyney M, Leeman LM. Planned Home VBAC in the United States, 2004-2009: Outcomes, Maternity Care Practices, and Implications for Shared Decision Making. Birth. 2015;42(4):299-308. doi: https://dx.doi.org/10.1111/birt.12188. | Wrong population (not only women with normal pregnancies and expected normal births) |
| Cox KJ, Schlegel R, Payne P, Teaf D, Albers L. Outcomes of planned home births attended by certified nurse-midwives in southeastern Pennsylvania, 1983-2008. J Midwifery Womens Health. 2013;58(2):145-9. doi: https://dx.doi.org/10.1111/j.1542-2011.2012.00217.x. | Wrong population (not only women with normal pregnancies and expected normal births) |
| Cross-Sudworth F, Hindley J, Cheatham C, Clarke P, McAree T. Creating a dedicated homebirth service: Results of a 3-year pilot. Br J Midwifery. 2018;26(3):164-70. doi: doi:10.12968/bjom.2018.26.3.164. | Wrong population (not only women with normal pregnancies and expected normal births) |
| Darling EK, Bennett N, Burton N, Marquez O. Outcomes of uninsured midwifery clients in Ontario, Canada: A retrospective cohort study. Midwifery. 2019a;77:24-31. doi: https://dx.doi.org/10.1016/j.midw.2019.06.009. | Wrong focus (comparison insured-uninsured women) |
| Darling EK, Lawford KMO, Wilson K, Kryzana Uskas M, Bourgeault IL. Distance from Home Birth to Emergency Obstetric Services and Neonatal Outcomes: A Cohort Study. J Midwifery Womens Health. 2019b;64:170-8. doi: https://dx.doi.org/10.1111/jmwh.12896. | Wrong population (includes w 42+) (case series) |
| de Jonge A, Mesman JA, Mannien J, Zwart JJ, van Dillen J, van Roosmalen J. Severe adverse maternal outcomes among low risk women with planned home versus hospital births in the Netherlands: nationwide cohort study. BMJ. 2013;346:f3263. doi: https://dx.doi.org/10.1136/bmj.f3263. | Wrong comparison (AMU) |
| de Jonge A, Peters L, Geerts CC, van Roosmalen JJM, Twisk JWR, Brocklehurst P, et al. Mode of birth and medical interventions among women at low risk of complications: A cross-national comparison of birth settings in England and the Netherlands. PLoS ONE [Electronic Resource]. 2017;12(7):e0180846. doi: https://dx.doi.org/10.1371/journal.pone.0180846. | Wrong comparison (comparison between different countries) |
| de Jonge A, van der Goes BY, Ravelli AC, Amelink-Verburg MP, Mol BW, Nijhuis JG, et al. Perinatal mortality and morbidity in a nationwide cohort of 529,688 low-risk planned home and hospital births. BJOG. 2009;116(9):1177-84. doi: https://dx.doi.org/10.1111/j.1471-0528.2009.02175.x. | Duplicate data with de Jonge, 2015 |
| Dencker A, Smith V, McCann C, Begley C. Midwife-led maternity care in Ireland - a retrospective cohort study. BMC Pregnancy Childbirth. 2017;17(1):101. doi: https://dx.doi.org/10.1186/s12884-017-1285-9. | Wrong intervention (in hospital) |
| Dixon L, Fletcher L, Tracy S, Guilliland K, Pairman S, Hendry C. Midwives care during the Third Stage of Labour: an analysis of the New Zealand College of Midwives Midwifery Database 2004-2008 [corrected] [published erratum appears in J N Z Coll Midwives 2010 May;42:6]. J N Z Coll Midwives. 2009(41):20-5. | Wrong intervention (management of third stage of labour) |
| Dixon L, Tracy SK, Guilliland K, Fletcher L, Hendry C, Pairman S. Outcomes of physiological and active third stage labour care amongst women in New Zealand. Midwifery. 2013;29(1):67-74. doi: https://dx.doi.org/10.1016/j.midw.2011.11.003 | Wrong focus (physiologic vs active management) |
| Dixon L, Prileszky G, Guillilan K, Miller S, Anderson J. Place of birth and outcomes for a cohort of low risk women in New Zealand: A comparison with Birthplace England. J N Z Coll Midwives. 2014(50):11-8. doi: 10.12784/nzcomjn150.2014.2.11-18. | Wrong population (not only women with normal pregnancies and expected normal births) |
| Edqvist M, Blix E, Hegaard HK, Olafsdottir O, Hildingsson I, Ingversen K, et al. Perineal injuries and birth positions among 2992 women with a low risk pregnancy who opted for a homebirth. BMC Pregnancy Childbirth. 2016;16. doi: https://dx.doi.org/10.1186/s12884-016-0990-0. | Wrong population (not specified if only low-risk women according to the PICO were included) |
| Edwards J. Reclaiming a home birth -- midwives supporting women. Midwifery Matters. 2009(122):13-4. | Wrong study design (case report, not mortality, not transfer) |
| Elder HR, Alio AP, Fisher SG. Investigating the debate of home birth safety: A critical review of cohort studies focusing on selected infant outcomes. Jpn J Nurs Sci. 2016;13(3):297-308. doi: https://dx.doi.org/10.1111/jjns.12116. | Wrong study design (review, not systematic) |
| Elkington M, Kurinczuk JJ, Pasupathy D, Plachcinski R, Rogers J, Williams C, et al. Postpartum haemorrhage occurring in UK midwifery units: A national population-based case-control study to investigate incidence, risk factors and outcomes. PLoS ONE [Electronic Resource]. 2023;18(10):e0291795. doi: https://dx.doi.org/10.1371/journal.pone.0291795. | Wrong focus (risk factors for transfer for postpartum haemorrhage)? |
| Erickson EN, Bovbjerg ML, Cheyney MJ. Factors affecting third-stage management and postpartum hemorrhage in planned midwife-led home and birth center births in the United States. Birth. 2020;47(4):397-408. doi: https://dx.doi.org/10.1111/birt.12497. | Wrong population (not only women with normal pregnancies and expected normal births) |
| Evers AC, Brouwers HA, Hukkelhoven CW, Nikkels PG, Boon J, van Egmond-Linden A, et al. Perinatal mortality and severe morbidity in low and high risk term pregnancies in the Netherlands: prospective cohort study. BMJ. 2010;341:c5639. doi: https://dx.doi.org/10.1136/bmj.c5639. | Wrong population (not only women with normal pregnancies and expected normal births) |
| Fahy K, Hastie C, Bisits A, Marsh C, Smith L, Saxton A. Holistic physiological care compared with active management of the third stage of labour for women at low risk of postpartum haemorrhage: a cohort study. Women Birth. 2010;23(4):146-52. doi: https://dx.doi.org/10.1016/j.wombi.2010.02.003. | Wrong focus (third stage of labour) |
| Ford J, Pett G. 'BBA' birth: analysis of one year's 'born before arrived' births (n=29) and trends in BBA birth 2000-07 in a large English maternity ward. MIDIRS Midwifery Digest. 2008;18(2):217-23. | Wrong intervention (mixed attended/unattended) |
| Galkova G, Bohm P, Hon Z, Herman T, Doubrava R, Navratil L. Comparison of Frequency of Home Births in the Member States of the EU Between 2015 and 2019. Glob Pediatr Health. 2022;9. doi: https://dx.doi.org/10.1177/2333794X211070916. | Wrong outcome (case series, not mortality or transfer) |
| Gao Y, Wilkes L, Tafe A, Quanchi A, Ruthenberg L, Warriner M, et al. Clinical outcomes and financial estimates for women attending the largest private midwifery service in Australia compared to national data: a retrospective cohort study. Women Birth. 2024;37(3):101591. doi: https://dx.doi.org/10.1016/j.wombi.2024.101591. | Wrong population (not only women with normal pregnancies and expected normal births) |
| Gaudineau A, Sauleau EA, Nisand I, Langer B. Obstetric and neonatal outcomes in a home-like birth centre: a case-control study. Arch Gynecol Obstet. 2013;287(2):211-6. doi: 10.1007/s00404-012-2553-6. | Wrong intervention (in hospital) |
| Goh AH, Kapoor DA, Nguyen A, Soman D, Jolles DR. Characteristics and Outcomes Among Asian Birthing People in the American Association of Birth Centers Perinatal Data Registry. Birth: Issues in Perinatal Care. 2026;53(1):81-7. doi: 10.1111/birt.12923 | Wrong study design (comparison Asian and White women, mortality or transfer not reported) |
| Granger Howard ME, Phibbs CS, Lorch S, Passarella M, Boghossian NS. Planned Community Birth and Birth Outcomes. JAMA Pediatrics. 2026;180(2):160-70. doi: 10.1001/jamapediatrics.2025.4840. | Wrong intervention (midwife certification not obligatory in the beginning of the study period) and wrong population (previous caesarean sections included) |
| Gravesteijn BY, Boderie NW, van den Akker T, Bertens LCM, Bloemenkamp K, Burgos Ochoa L, et al. Effect of COVID-19 lockdown on maternity care and maternal outcome in the Netherlands: a national quasi-experimental study. Public Health. 2024;235:15-25. doi:https://doi.org/10.1016/j.puhe.2024.06.024. | Wrong focus (Covid-19 lockdown) |
| Grigg CP, Tracy SK, Tracy M, Daellenbach R, Kensington M, Monk A, et al. Evaluating Maternity Units: a prospective cohort study of freestanding midwife-led primary maternity units in New Zealand-clinical outcomes. BMJ Open. 2017;7(8):e016288. doi: https://dx.doi.org/10.1136/bmjopen-2017-016288. | Wrong population (not only women with normal pregnancies and expected normal births) |
| Grunebaum A, McCullough LB, Arabin B, Brent RL, Levene MI, Chervenak FA. Neonatal Mortality of Planned Home Birth in the United States in Relation to Professional Certification of Birth Attendants. PLoS ONE [Electronic Resource]. 2016;11(5):e0155721. doi: https://dx.doi.org/10.1371/journal.pone.0155721. | Wrong population (not only women with normal pregnancies and expected normal births) |
| Grunebaum A, McCullough LB, Arabin B, Dudenhausen J, Orosz B, Chervenak FA. Underlying causes of neonatal deaths in term singleton pregnancies: home births versus hospital births in the United States. J Perinat Med. 2017a;45(3):349-57. doi: https://dx.doi.org/10.1515/jpm-2016-0200. | Wrong population (not only women with normal pregnancies and expected normal births) |
| Grunebaum A, McCullough LB, Bornstein E, Lenchner E, Katz A, Spiryda LB, et al. Neonatal outcomes of births in freestanding birth centers and hospitals in the United States, 2016-2019. Am J Obstet Gynecol. 2022;226(1):116.e1-.e7. doi: https://dx.doi.org/10.1016/j.ajog.2021.06.093. | Wrong intervention (not ICM certified midwifes) |
| Grunebaum A, McCullough LB, Brent RL, Arabin B, Levene MI, Chervenak FA. Justified skepticism about Apgar scoring in out-of-hospital birth settings. J Perinat Med. 2015a;43(4):455-60. doi: https://dx.doi.org/10.1515/jpm-2014-0003. | Wrong population (not only women with normal pregnancies and expected normal births) |
| Grunebaum A, McCullough LB, Brent RL, Arabin B, Levene MI, Chervenak FA. Perinatal risks of planned home births in the United States. Am J Obstet Gynecol. 2015b;212(3):350.e1-6. doi: https://dx.doi.org/10.1016/j.ajog.2014.10.021. | Wrong focus (frequency of risk factors) |
| Grunebaum A, McCullough LB, Brent RL, Arabin B, Levene MI, Chervenak FA. Perinatal risks of planned home births in the United States. Obstet Gynecol Surv. 2015c;70:428-30. doi: https://dx.doi.org/10.1097/01.ogx.0000469200.37361.b3. | Wrong publication type (editorial) |
| Grunebaum A, McCullough LB, Chervenak FA, Dudenhausen JW. Sudden severe fetal compromise at a planned home birth - A case of umbilical cord prolapse. Case Rep Perinat Med. 2019;8. | Wrong population (not only women with normal pregnancies and normal expected births) |
| Grunebaum A, McCullough LB, Orosz B, Chervenak FA. Neonatal mortality in the United States is related to location of birth (hospital versus home) rather than the type of birth attendant. Am J Obstet Gynecol. 2020;223(2):254.e1-.e8. doi: https://dx.doi.org/10.1016/j.ajog.2020.01.045. | Wrong population (not only women with normal pregnancies and expected normal births) |
| Grunebaum A, McCullough LB, Sapra KJ, Arabin B, Chervenak FA. Planned home births: the need for additional contraindications. Am J Obstet Gynecol. 2017b;216(4):401.e1-.e8. doi: https://dx.doi.org/10.1016/j.ajog.2017.01.012. | Wrong population (not only women with normal pregnancies and expected normal births) |
| Grunebaum A, McCullough LB, Sapra KJ, Brent RL, Levene MI, Arabin B, et al. Apgar score of 0 at 5 minutes and neonatal seizures or serious neurologic dysfunction in relation to birth setting. Am J Obstet Gynecol. 2013;209(4):323.e1-6. doi: https://dx.doi.org/10.1016/j.ajog.2013.06.025. | Wrong population (not only women with normal pregnancies and normal expected births) |
| Grunebaum A, McCullough LB, Sapra KJ, Brent RL, Levene MI, Arabin B, et al. Apgar score of 0 at 5 minutes and neonatal seizures or serious neurologic dysfunction in relation to birth setting. Obstet Gynecol Surv. 2014a;69:67-9. doi: https://dx.doi.org/10.1097/01.ogx.0000444673.43584.32. | Wrong publication type (editorial) |
| Grunebaum A, McCullough LB, Sapra KJ, Brent RL, Levene MI, Arabin B, et al. Early and total neonatal mortality in relation to birth setting in the United States, 2006-2009. Am J Obstet Gynecol. 2014b;211(4):390.e1-7. doi: https://dx.doi.org/10.1016/j.ajog.2014.03.047. | Wrong population (not only women with normal pregnancies and normal expected births) |
| Gutteridge K. Midwifery-led care for a low-risk cohort -- a clinical outcomes overview: over a three year period in a multicultural setting. MIDIRS Midwifery Digest. 2015;25(2):175-85. | Wrong population (study population not clearly defined) |
| Halfdansdottir B, Ellinger-Kaya K, Fjosne K, Lindgren H, Hegaard HK, Blix E. The association between waterbirth and perineal injury or other adverse outcomes among low-risk women with physiological birth: Results from the Nordic Home Birth Cohort Study. Women Birth. 2024;37(4):101625. doi: https://dx.doi.org/10.1016/j.wombi.2024.101625. | Wrong population (not only women with normal pregnancies and expected normal births) |
| Halfdansdottir B, Hildingsson I, Smarason AK, Sveinsdottir H, Olafsdottir OA. Contraindications in planned home birth in Iceland: A retrospective cohort study. Sex Reprod Healthc. 2018;15:10-7. doi: https://dx.doi.org/10.1016/j.srhc.2017.11.002. | Wrong population (not only women with normal pregnancies and expected normal births) |
| Halfdansdottir B, Smarason AK, Olafsdottir OA, Hildingsson I, Sveinsdottir H. Outcome of planned home and hospital births among low-risk women in Iceland in 2005-2009: a retrospective cohort study. Birth. 2015;42(1):16-26. doi: https://dx.doi.org/10.1111/birt.12150. | Wrong population (not only women with normal pregnancies and expected normal births) |
| Hansel S, Kuyateh MH, Bello-Ogunu F, Stranton DT, Hicks K, Huber LRB. Associations between Place of Birth, Type of Attendant, and Small for Gestational Age Births among Pregnant non-Hispanic Black Medicaid Recipients. J Midwifery Womens Health. 2022;67(2):202-8. doi: https://dx.doi.org/10.1111/jmwh.13312. | Wrong focus (associations between small for gestational age and different factors) |
| Hays K, Denmark M, Levine A, de Regt RH, Andersen HF, Weiss K. Smooth Transitions: Enhancing Interprofessional Collaboration when Planned Community Births Transfer to Hospital Care. J Midwifery Womens Health. 2022;67(6):701-6. doi: https://dx.doi.org/10.1111/jmwh.13441. | Wrong focus (description of a program) |
| Hendrix MJ, Evers SM, Basten MC, Nijhuis JG, Severens JL. Cost analysis of the Dutch obstetric system: low-risk nulliparous women preferring home or short-stay hospital birth--a prospective non-randomised controlled study. BMC Health Serv Res. 2009;9:211. doi: https://dx.doi.org/10.1186/1472-6963-9-211. | Cost analysis. Incorrect control group (AMU) |
| Heron J, Haque S, Oyebode F, Craddock N, Jones I. A longitudinal study of hypomania and depression symptoms in pregnancy and the postpartum period. Bipolar Disord. 2009;11(4):410-7. doi: https://dx.doi.org/10.1111/j.1399-5618.2009.00685.x. | Wrong population (not only women with normal pregnancies and expected normal births) |
| Hickson C, Hoskins F, Ogollah R, Walker KF, Thornton JG. The risks of a range of maternal pregnancy choices, expressed as "baby micromorts" (risk of death per million births). Eur J Obstet Gynecol Reprod Biol. 2020;251:194-8. doi: https://dx.doi.org/10.1016/j.ejogrb.2020.05.051. | Wrong study design (review, not systematic) |
| Hildingsson I, Blix E, Hegaard H, Huitfeldt A, Ingversen K, Olafsdottir OA, et al. How Long Is a Normal Labor? Contemporary Patterns of Labor and Birth in a Low-Risk Sample of 1,612 Women from Four Nordic Countries. Birth. 2015;42(4):346-53. doi: https://dx.doi.org/10.1111/birt.12191. | Wrong population, wrong outcome (case series, not mortality, transferred women with instrumental birth excluded) |
| Hirschhorn-Edwards N, Dahlen H, Kildea S, Tracy SK. Are Private Obstetric Services Safer and More Cost Effective Than Midwife-Led Care in Australia? BJOG: An International Journal of Obstetrics and Gynaecology. 2025. doi: https://dx.doi.org/10.1111/1471-0528.18365. | Wrong publication type (letter) |
| Hitzert M, Hermus MM, Boesveld, II, Franx A, van der Pal-de Bruin KK, Steegers EE, et al. Cost-effectiveness of planned birth in a birth centre compared with alternative planned places of birth: results of the Dutch Birth Centre study. BMJ Open. 2017;7(9):e016960. doi: https://dx.doi.org/10.1136/bmjopen-2017-016960. | Cost analysis. Insufficient methodology |
| Hodgkin K, Joshy G, Lokuge K. Do Indonesian midwifery-led birth units provide safe, accessible care? A secondary analysis of demographic health survey cross-sectional data. Sexual & Reproductive HealthCare. 2025;44:N.PAG-N.PAG. doi: 10.1016/j.srhc.2025.101089 | Wrong population (not high-income setting) |
| Hoehn-Velasco L, Ross L, Phillippi RD, Niemczyk NA, Cammarano D, Calvin S, et al. Neonatal morbidity and mortality in birth centers in the United States 2018-2021: An observational study of low-risk birthing individuals. Birth. 2024;51(3):659-66. doi: 10.1111/birt.12823. | Wrong population (diabetes included, no information regarding previous caesarean sections), wrong intervention (physician could be birth attendant in FMUs) |
| Hoehn-Velasco L, Ross L, Phillippi RD, Niemczyk NA, Cammarano D, Calvin S, et al. Neonatal Morbidity and Mortality in Birth Centers in the United States 2018-2021: An Observational Study of Low-Risk Birthing Individuals. Obstet Gynecol Surv. 2025;80(3):146-7. doi: https://dx.doi.org/10.1097/01.ogx.0001110348.28933.85. | Wrong publication type (letter) |
| Hollowell J, Li Y, Bunch K, Brocklehurst P. A comparison of intrapartum interventions and adverse outcomes by parity in planned freestanding midwifery unit and alongside midwifery unit births: secondary analysis of 'low risk' births in the birthplace in England cohort. BMC Pregnancy Childbirth. 2017;17(1):95. doi: https://dx.doi.org/10.1186/s12884-017-1271-2. | Wrong comparison (different midwife units) |
| Hollowell J, Rowe R, Townend J, Knight M, Li Y, Linsell L, et al. The Birthplace in England national prospective cohort study: further analyses to enhance policy and service delivery decision-making for planned place of birth. NIHR Journals Library Health Services and Delivery Research2015. 2015;8:08. doi: <https://dx.doi.org/10.3310/hsdr03360>. | Wrong study design (review, not systematic) |
| Homer CS, Thornton C, Scarf VL, Ellwood DA, Oats JJ, Foureur MJ, et al. Birthplace in New South Wales, Australia: an analysis of perinatal outcomes using routinely collected data. BMC Pregnancy Childbirth. 2014;14:206. doi: https://dx.doi.org/10.1186/1471-2393-14-206. | Wrong population (not only women with normal pregnancies and expected normal births) |
| Hoque AM, Buckus S, Hoque M. Incidence of post-partum complications and referrals of mothers and neonates to hospitals from a Midwife Obstetric Unit. Afr Health Sci. 2024;24:243-54. doi: doi:https://doi.org/10.4314/ahs.v24i2.27. | Wrong population (not high-income setting) |
| Hu Y, Allen J, Ellwood D, Slavin V, Gamble J, Toohill J, et al. The financial impact of offering publicly funded homebirths: A population-based microsimulation in Queensland, Australia. Women Birth. 2024;37(1):137-43. doi: https://dx.doi.org/10.1016/j.wombi.2023.07.129. | Cost analysis. Hypothetical cost model, no data Excluded. |
| Hutton EK, Cappelletti A, Reitsma AH, Simioni J, Horne J, McGregor C, et al. Outcomes associated with planned place of birth among women with low-risk pregnancies. CMAJ. 2016a;188(5):E80-E90. doi: https://dx.doi.org/10.1503/cmaj.150564. | Wrong population (not only women with normal pregnancies and expected normal births) |
| Hutton EK, Cappelletti A, Reitsma AH, Simioni J, Horne J, McGregor C, et al. Outcomes Associated With Planned Place of Birth Among Women With Low-Risk Pregnancies. Obstet Gynecol Surv. 2016b;71:205-6. doi: https://dx.doi.org/10.1097/01.OGX.0000481815.84386.6D. | Wrong publication type (editorial) |
| Hutton EK, Reitsma A, Simioni J, Brunton G, Kaufman K. Perinatal or neonatal mortality among women who intend at the onset of labour to give birth at home compared to women of low obstetrical risk who intend to give birth in hospital: A systematic review and meta-analyses. EClinicalMedicine. 2019;14:59-70. doi: https://dx.doi.org/10.1016/j.eclinm.2019.07.005. | Wrong population (not only women with normal pregnancies and expected normal births) |
| Hutton EK, Reitsma AH, Kaufman K. Outcomes associated with planned home and planned hospital births in low-risk women attended by midwives in Ontario, Canada, 2003-2006: a retrospective cohort study. Birth. 2009;36(3):180-9. doi: https://dx.doi.org/10.1111/j.1523-536X.2009.00322.x. | Wrong population (not only women with normal pregnancies and expected normal births) |
| Ignatov T, Eggemann H, Costa S, Ignatov A, Costa SD. Perinatal and maternal outcomes at term in low-risk pregnancies according to NICE criteria: comparison between a tertiary obstetrical hospital and midwife-attended units. Arch Gynecol Obstet. 2017;296(2):223-9. doi: 10.1007/s00404-017-4423-8. | Wrong design (comparison to historical cohort) |
| Janssen PA, Mitton C, Aghajanian J. Costs of Planned Home vs. Hospital Birth in British Columbia Attended by Registered Midwives and Physicians. PLoS ONE [Electronic Resource]. 2015;10(7):e0133524. doi: https://dx.doi.org/10.1371/journal.pone.0133524. | Cost analysis. Incorrect population, includes caesarean section |
| Janssen PA, Saxell L, Page LA, Klein MC, Liston RM, Lee SK. Outcomes of planned home birth with registered midwife versus planned hospital birth with midwife or physician. CMAJ. 2009;181(6):377-83. doi: https://dx.doi.org/10.1503/cmaj.081869. | Wrong population (not only women with normal pregnancies and expected normal births) |
| Jensen S, Colmorn LB, Schroll AM, Krebs L. Quality assessment of home births in Denmark. Dan Med J. 2017;64(5). | Wrong intervention (mixed planned/unplanned home birth) |
| Johansen LT, Oian P. Neonatal deaths and injuries. Tidsskr Nor Laegeforen. 2011;131:2465-8. doi: https://doi.org/10.4045/tidsskr.11.0775 | Wrong focus (risk factors) |
| Johnson KC, Daviss B. Outcomes of planned home births with certified professional midwives: large prospective study in North America. BMJ. 2005;330(7505):1416-9. doi: 10.1136/bmj.330.7505.1416. | Wrong population (not only women with normal pregnancies and expected normal births) |
| Jolles DR, Niemczyk N, Hoehn Velasco L, Wallace J, Wright J, Stapleton S, et al. The birth center model of care: Staffing, business characteristics, and core clinical outcomes. Birth. 2023;50(4):1045-56. doi: https://dx.doi.org/10.1111/birt.12745. | Wrong focus (birth centre staffing models) |
| Kallianidis A, Smit M, Van RJ. Shoulder dystocia in primary midwifery care in the Netherlands. Acta Obstet Gynecol Scand. 2016;95(2):203‐9. doi: 10.1111/aogs.12800. | Wrong population (not only women with normal pregnancies and expected normal births) |
| Kataoka Y, Eto H, Iida M. Outcomes of independent midwifery attended births in birth centres and home births: a retrospective cohort study in Japan. Midwifery. 2013;29(8):965-72. doi: https://dx.doi.org/10.1016/j.midw.2012.12.020. | Wrong population (not only women with normal pregnancies and expected normal births) |
| Kataoka Y, Masuzawa Y, Kato C, Eto H. Maternal and neonatal outcomes in birth centers versus hospitals among women with low-risk pregnancies in Japan: A retrospective cohort study. Jpn J Nurs Sci. 2018;15(1):91-6. doi: https://dx.doi.org/10.1111/jjns.12171. | Wrong population (not only women with normal pregnancies and expected normal births) |
| Kennare RM, Keirse MJ, Tucker GR, Chan AC. Planned home and hospital births in South Australia, 1991-2006: differences in outcomes. Med J Aust. 2010;192(2):76-80. doi: https://dx.doi.org/10.5694/j.1326-5377.2010.tb03422.x. | Wrong population (not only women with normal pregnancies and expected normal births) |
| Kenny C, Devane D, Normand C, Clarke M, Howard A, Begley C. A cost-comparison of midwife-led compared with consultant-led maternity care in Ireland (the MidU study). Midwifery. 2015;31(11):1032-8. doi: https://dx.doi.org/10.1016/j.midw.2015.06.012. | Wrong intervention (in hospital) |
| Kildea S, Gao Y, Rolfe M, Josif CM, Bar-Zeev SJ, Steenkamp M, et al. Remote links: Redesigning maternity care for Aboriginal women from remote communities in Northern Australia - A comparative cohort study. Midwifery. 2016;34:47-57. doi: https://dx.doi.org/10.1016/j.midw.2016.01.009. | Wrong population (not only women with normal pregnancies and expected normal births) |
| Kobayashi S, Hanada N, Matsuzaki M, Takehara K, Ota E, Sasaki H, et al. Assessment and support during early labour for improving birth outcomes. Cochrane Database Syst Rev. 2017;4:CD011516. doi: https://dx.doi.org/10.1002/14651858.CD011516.pub2. | Wrong intervention (assessment and support interventions) |
| Krepelka P, Herman H, Velebil P, Mechurova A, Hanacek J, Stranak Z, et al. Complications of planned home births in the Czech Republic. Ginekol Pol. 2023;17:17. doi: https://dx.doi.org/10.5603/GP.a2023.0001. | Wrong intervention (mixed midwife attended/unattended) |
| Krepelka P, Velebil P, Mechurova A, Stranak Z, Feyereisl J. Complications of planned home births in the Czech Republic between 2016-2017. Cent Eur J Public Health. 2020;28(3):230-6. doi: https://dx.doi.org/10.21101/cejph.a5641. | Wrong intervention (mixed midwife attended/unattended) |
| Laws PJ, Tracy SK, Sullivan EA. Perinatal outcomes of women intending to give birth in birth centers in Australia. Birth. 2010;37(1):28-36. doi: 10.1111/j.1523-536X.2009.00375.x. | Wrong population (not only women with normal pregnancies and expected normal births) |
| Li Q, Jenkins D, Kinsman S. Birth Settings and the Validation of Neonatal Seizures Recorded in Birth Certificates Compared to Medicaid Claims and Hospital Discharge Abstracts Among Live Births in South Carolina, 1996-2013. Matern Child Health J. 2017;21(5):1047-54. doi: 10.1007/s10995-016-2200-0. | Wrong population (not only women with normal pregnancies and expected normal births) |
| Li Y, Townend J, Rowe R, Knight M, Brocklehurst P, Hollowell J. The effect of maternal age and planned place of birth on intrapartum outcomes in healthy women with straightforward pregnancies: secondary analysis of the Birthplace national prospective cohort study. BMJ Open. 2014;4(1):e004026. doi: https://dx.doi.org/10.1136/bmjopen-2013-004026. | Duplicate data with Brocklehurst, 2011 |
| Lindgren HE, Hildingsson IM, Christensson K, Radestad IJ. Transfers in planned home births related to midwife availability and continuity: a nationwide population-based study. Birth. 2008b;35(1):9-15. doi: doi:https://dx.doi.org/10.1111/j.1523-536X.2007.00206.x. | Wrong population (not only women with normal pregnancies and expected normal births |
| Lukasse M, Oian P, Aamodt G. [A midwife-led birthing unit]. Tidsskr Nor Laegeforen. 2006;126(2):170-2. | Wrong intervention (in hospital) |
| Lukasse M, Rowe R, Townend J, Knight M, Hollowell J. Immersion in water for pain relief and the risk of intrapartum transfer among low risk nulliparous women: secondary analysis of the Birthplace national prospective cohort study. BMC Pregnancy Childbirth. 2014;14:60. doi: https://dx.doi.org/10.1186/1471-2393-14-60. | Wrong comparison (different midwife units) |
| Lundeen T. Intrapartum and Postpartum Transfers to a Tertiary Care Hospital from Out-of-Hospital Birth Settings: A Retrospective Case Series. J Midwifery Womens Health. 2016;61(2):242-8. doi: https://dx.doi.org/10.1111/jmwh.12447. | Wrong population (not only women with normal pregnancies and expected normal births) |
| Macfarlane A, Dattani N, Gibson R, Harper G, Martin P, Scanlon M, et al. Births and their outcomes by time, day and year: a retrospective birth cohort data linkage study. Health Serv Deliv Res 2019;5(05). doi: https://doi.org/10.3310/hsdr07180 | Wrong focus (time, day and year) |
| Malloy MH. Infant outcomes of certified nurse midwife attended home births: United States 2000 to 2004. J Perinatol. 2010;30(9):622-7. doi: https://dx.doi.org/10.1038/jp.2010.12. | Wrong population (not only women with normal pregnancies and expected normal births) |
| Miller S, Skinner J. Are first-time mothers who plan home birth more likely to receive evidence-based care? A comparative study of home and hospital care provided by the same midwives. Birth. 2012;39(2):135-44. doi: https://dx.doi.org/10.1111/j.1523-536X.2012.00534.x. | Wrong study design (survey study) |
| Molina Perez A. Holistic care in midwifery-led birthing units. MIDIRS Midwifery Digest. 2020;30(1):66-70. | Wrong intervention (mixed in/out of hospital) |
| Monk A, Tracy M, Foureur M, Grigg C, Tracy S. Evaluating Midwifery Units (EMU): a prospective cohort study of freestanding midwifery units in New South Wales, Australia. BMJ Open. 2014;4(10):e006252. doi: https://dx.doi.org/10.1136/bmjopen-2014-006252. | Wrong population (not only women with normal pregnancies and expected normal births) |
| Monk AR, Grigg CP, Foureur M, Tracy M, Tracy SK. Freestanding midwifery units: Maternal and neonatal outcomes following transfer. Midwifery. 2017;46:24-8. doi: https://dx.doi.org/10.1016/j.midw.2017.01.006. | Wrong outcome (case series, not mortality) |
| Murakami M, Kawasaki H, Tagawa K, Maehara E, Tanaka M, Takashima M, et al. Midwife-Led Home Births in Japan: A 25-Year Retrospective Analysis of Care in Accordance with WHO Recommendations Before and After COVID-19. Healthcare (2227-9032). 2026;14(6):818. doi: https://dx.doi.org/10.3390/healthcare14060818 | Wrong study design (comparison outcomes before and during the pandemic, transfers are excluded) |
| Nethery E, Gordon W, Bovbjerg ML, Cheyney M. Rural community birth: Maternal and neonatal outcomes for planned community births among rural women in the United States, 2004-2009. Birth. 2018;45(2):120-9. doi: https://dx.doi.org/10.1111/birt.12322. | Wrong population and intervention (case series, not mortality; not all midwives certified) |
| Nethery E, Schummers L, Levine A, Caughey AB, Souter V, Gordon W. Birth Outcomes for Planned Home and Licensed Freestanding Birth Center Births in Washington State. Obstet Gynecol. 2021;138(5):693-702. doi: https://dx.doi.org/10.1097/AOG.0000000000004578. | Wrong comparison (different midwife units) |
| Nguyen UDT, Rothman KJ, Demissie S, Jackson DJ, Lang JM, Ecker JL. Epidural analgesia and risks of cesarean and operative vaginal deliveries in nulliparous and multiparous women. Matern Child Health J. 2010;14(5):705-12. doi: https://dx.doi.org/10.1007/s10995-009-0515-9. | Wrong focus (epidural analgesia) |
| Nguyen USDT, Rothman KJ, Demissie S, Jackson DJ, Lang JM, Ecker JL. Transfers Among Women Intending A Birth Center Delivery in the San Diego Birth Center Study. J Midwifery Womens Health. 2009;54:104-10. doi: doi:https://dx.doi.org/10.1016/j.jmwh.2008.11.002. | Wrong population (not only women with normal pregnancies and expected normal births) |
| Norum J, Heyd A, Hjelseth B, Svee T, Murer FA, Erlandsen R, et al. Quality of obstetric care in the sparsely populated sub-arctic area of Norway 2009-2011. BMC Pregnancy Childbirth. 2013;13:175. doi: https://dx.doi.org/10.1186/1471-2393-13-175. | Wrong comparison (different midwife units) |
| Nove A, Berrington A, Matthews Z. Comparing the odds of postpartum haemorrhage in planned home birth against planned hospital birth: results of an observational study of over 500,000 maternities in the UK. BMC Pregnancy Childbirth. 2012;12:130. doi: https://dx.doi.org/10.1186/1471-2393-12-130. | Wrong population (not only women with normal pregnancies and expected normal births) |
| O'Leary BD, Ciprike V. Are women attending a midwifery-led birthing center at increased risk of anal sphincter injury? Int Urogynecol J. 2020;31(3):583-9. doi: https://dx.doi.org/10.1007/s00192-019-04218-y. | Wrong intervention (in hospital) |
| Offerhaus P, Jans S, Hukkelhoven C, de Vries R, Nieuwenhuijze M. Women's characteristics and care outcomes of caseload midwifery care in the Netherlands: a retrospective cohort study. BMC Pregnancy Childbirth. 2020;20(1):517. doi: https://dx.doi.org/10.1186/s12884-020-03204-3. | Wrong focus (caseload midwifery) |
| Offerhaus PM, de Jonge A, van der Pal-de Bruin KM, Hukkelhoven CW, Scheepers PL, Lagro-Janssen AL. Change in primary midwife-led care in the Netherlands in 2000-2008: a descriptive study of caesarean sections and other interventions among 789,795 low risk births. Midwifery. 2014;30(5):560-6. doi: https://dx.doi.org/10.1016/j.midw.2013.06.013. | Wrong outcome (variation in intervention rates among midwifery practices) |
| Oian P, Askeland OM, Engelund IE, Roland B, Ebbing M. Births in Norwegian midwife-led birth units 2008-10; a population-based study. Tidsskr Nor Laegeforen. 2018;138(10):12. doi: https://dx.doi.org/10.4045/tidsskr.17.0842 | Wrong population (not only women with normal pregnancies and expected normal births) |
| Okui T, Nakashima N. Sociodemographic characteristics of non-institutional births and the association with neonatal and infant mortality in Japan. J Gynecol Obstet Hum Reprod. 2024;53(10):102834. doi: doi:https://doi.org/10.1016/j.jogoh.2024.102834. | Wrong intervention (mixed midwife led/physician led) |
| Olsen O, Clausen J. Planned hospital birth compared with planned home birth for pregnant women at low risk of complications. Cochrane Database Syst Rev. 2023(3). doi: 10.1002/14651858.CD000352.pub3. | Wrong publication type (systematic review |
| Oude Alink MB, Moors XRJ, de Jonge RCJ, Hartog DD, Houmes RJ, Stolker RJ. Prehospital Management of Peripartum Neonatal Complications by Helicopter Emergency Medical Service in the South West of the Netherlands: An Observational Study. Air Med J. 2020;39(6):489-93. doi: https://dx.doi.org/10.1016/j.amj.2020.08.002. | Wrong population (not only women with normal pregnancies and expected normal births) |
| Ovaskainen K, Ojala R, Gissler M, Luukkaala T, Tammela O. Is birth out-of-hospital associated with mortality and morbidity by seven years of age? PLoS ONE [Electronic Resource]. 2021;16(4):e0250163. doi: https://dx.doi.org/10.1371/journal.pone.0250163. | Wrong population (not only women with normal pregnancies and expected normal births) |
| Ovaskainen K, Ojala R, Tihtonen K, Gissler M, Luukkaala T, Tammela O. Planned home deliveries in Finland, 1996-2013. J Perinatol. 2019;39(2):220-8. doi: https://dx.doi.org/10.1038/s41372-018-0267-8. | Wrong population (not only women with normal pregnancies and expected normal births) |
| Overgaard C, Fenger-Gron M, Sandall J. Freestanding midwifery units versus obstetric units: does the effect of place of birth differ with level of social disadvantage? BMC Public Health. 2012;12:478. doi: https://dx.doi.org/10.1186/1471-2458-12-478. | Wrong study design (overlap, secondary analysis of Overgaard 2011). |
| Overgaard C, Moller AM, Fenger-Gron M, Knudsen LB, Sandall J. Freestanding midwifery unit versus obstetric unit: a matched cohort study of outcomes in low-risk women. BMJ Open. 2011;1(2):e000262. doi: https://dx.doi.org/10.1136/bmjopen-2011-000262. | Wrong intervention (FMUs located in community hospitals with an ICU but without obstetric service |
| Palau-Costafreda R, Orus-Covisa L, Vicente-Castellvi E, Espada-Trespalacios X, Catala AM, Alcover C, et al. Impact of midwifery-led units in Spain: lessons from the first 5 years. Health Policy. 2025;162:105441. doi: https://dx.doi.org/10.1016/j.healthpol.2025.105441. | Wrong intervention (AMUs) |
| Papoutsis D, Antonakou A. Birth centres in Greece The role of the midwifery model of intrapartum care on the timing of births. Archives of Hellenic Medicine. 2026;43(3):408-14. | Wrong publication type (letter) |
| Park MR, Lee JY. Length of Stay, Health Care Cost, Postpartum Discomfort, and Satisfaction with Medical Service in Puerperas Giving Birth in Midwifery Clinic and Hospitals. Korean J Women Health Nurs. 2018;24(1):24-32. doi: https://dx.doi.org/10.4069/kjwhn.2018.24.1.24. | Wrong language (Korean) |
| Permezel M, Milne KJ. Pregnancy outcome at term in low-risk population: study at a tertiary obstetric hospital. J Obstet Gynaecol Res. 2015;41(8):1171-7. doi: https://dx.doi.org/10.1111/jog.12695. | Wrong intervention (in hospital) |
| Phillippi JC, Danhausen K, Alliman J, Phillippi RD. Neonatal Outcomes in the Birth Center Setting: A Systematic Review. J Midwifery Womens Health. 2018;63(1):68-89. doi: https://dx.doi.org/10.1111/jmwh.12701. | Wrong publication type (systematic review) |
| Pillai S, Cheyney M, Everson CL, Bovbjerg ML. Fetal macrosomia in home and birth center births in the United States: Maternal, fetal, and newborn outcomes. Birth. 2020;47(4):409-17. doi: https://dx.doi.org/10.1111/birt.12506. | Wrong population (not only women with normal pregnancies and expected normal births) |
| Potera C. A freestanding birthing center trumps hospitals. Am J Nurs. 2013;113(8):17. doi: https://dx.doi.org/10.1097/01.NAJ.0000432948.27150.80. | Wrong publication type (research summary) |
| Ravelli AC, Tromp M, van Huis M, Steegers EA, Tamminga P, Eskes M, et al. Decreasing perinatal mortality in The Netherlands, 2000-2006: a record linkage study. J Epidemiol Community Health. 2009;63(9):761-5. doi: https://dx.doi.org/10.1136/jech.2008.080440. | Wrong population (not only women with normal pregnancies and expected normal births) |
| Reitsma A, Simioni J, Brunton G, Kaufman K, Hutton EK. Maternal outcomes and birth interventions among women who begin labour intending to give birth at home compared to women of low obstetrical risk who intend to give birth in hospital: A systematic review and meta-analyses. EClinicalMedicine. 2020;21:100319. doi: https://dx.doi.org/10.1016/j.eclinm.2020.100319. | Wrong population (not only women with normal pregnancies and expected normal births) |
| Rollet C, Le Ray C, Vendittelli F, Blondel B, Chantry AA. Severe adverse maternal and neonatal outcomes according to the planned birth setting being midwife-led birth centers or obstetric-led units. Acta Obstet Gynecol Scand. 2024;103(12):2465-74. doi: doi:https://doi.org/10.1111/aogs.14971. | Wrong intervention (AMU) |
| Rossi AC, Prefumo F. Planned home versus planned hospital births in women at low-risk pregnancy: A systematic review with meta-analysis. Eur J Obstet Gynecol Reprod Biol. 2018;222:102-8. doi: https://dx.doi.org/10.1016/j.ejogrb.2018.01.016. | Wrong population (not only women with normal pregnancies and expected normal births) |
| Rowe R, Draper ES, Kenyon S, Bevan C, Dickens J, Forrester M, et al. Intrapartum-related perinatal deaths in births planned in midwifery-led settings in Great Britain: findings and recommendations from the ESMiE confidential enquiry. BJOG. 2020;127(13):1665-75. doi: https://dx.doi.org/10.1111/1471-0528.16327. | Wrong population (not only women with normal pregnancies and expected normal births) |
| Rowe R, Fitzpatrick R, Hollowell J, Kurinczuk J, Rowe RE, Fitzpatrick R, et al. Transfers of women planning birth in midwifery units: data from the birthplace prospective cohort study. BJOG. 2012;119(9):1081-90. doi: 10.1111/j.1471-0528.2012.03414.x. | Duplication with Rowe 2013 |
| Royds-Jones M. Birth centres are a safe option. MIDIRS Midwifery Digest. 2006;16(3):411-3. | Wrong population, wrong outcome (case series, not mortality) |
| Sarella A, Velli D, Kontoyannis M, Kokkosi E, Tzitiridou-Chatzopoulou M, Orovou E. Maternal and perinatal outcomes based on the planned birthplace for women with low-risk pregnancies: the German model. Review of Clinical Pharmacology and Pharmacokinetics, International Edition. 2025;39(1):43-50. doi: https://dx.doi.org/10.61873/JYAN4719. | Wrong intervention (AMU) |
| Scarf VL, Rossiter C, Vedam S, Dahlen HG, Ellwood D, Forster D, et al. Maternal and perinatal outcomes by planned place of birth among women with low-risk pregnancies in high-income countries: A systematic review and meta-analysis. Midwifery. 2018;62:240-55. doi: https://dx.doi.org/10.1016/j.midw.2018.03.024. | Wrong population (not only women with normal pregnancies and expected normal births) |
| Schildberger B, Riedmann M, Leitner H, Stelzl P. Obstetric and Neonatal Outcomes Following Hospital Transfers of Home Births and Births in Midwife-led Units in Austria. Geburtshilfe Frauenheilkd. 2023;84:264-73. doi: https://dx.doi.org/10.1055/a-2249-7228. | Wrong intervention (comparison of planned hospital with transferred home birth) |
| Schroeder L, Patel N, Keeler M, Rocca-Ihenacho L, Macfarlane AJ. The economic costs of intrapartum care in Tower Hamlets: A comparison between the cost of birth in a freestanding midwifery unit and hospital for women at low risk of obstetric complications. Midwifery. 2017;45:28-35. doi: https://dx.doi.org/10.1016/j.midw.2016.11.006. | Cost analysis. Compares birth center vs hospital, not home births; overlaps with 2012) |
| Seijmonsbergen-Schermers AE, Peerdeman KM, van den Akker T, Titulaer LM, Roovers JP, Peters LL, et al. Differences in rates of severe perineal trauma between midwife-led and obstetrician-led care in the Netherlands: A nationwide cohort study. Heliyon. 2024a;10(2):e24609. doi: https://dx.doi.org/10.1016/j.heliyon.2024.e24609. | Wrong intervention (mixed intervention) |
| Seijmonsbergen-Schermers AE, Peters LL, Jans S, Verhoeven CJ, de Jonge A. Decline in home births associated with faster increase in trend of postpartum haemorrhage and manual removal of the placenta. Sex Reprod Healthc. 2024b;40:100974. doi: https://dx.doi.org/10.1016/j.srhc.2024.100974. | Wrong population, outcome (case series, not mortality or transfer) |
| Seijmonsbergen-Schermers AE, Zondag DC, Nieuwenhuijze M, van den Akker T, Verhoeven CJ, Geerts CC, et al. Regional variations in childbirth interventions and their correlations with adverse outcomes, birthplace and care provider: A nationwide explorative study. PLoS ONE [Electronic Resource]. 2020;15(3):e0229488. doi: https://dx.doi.org/10.1371/journal.pone.0229488. | Wrong intervention (not led by obstetricians) |
| Shahtahmasebi Z. MY MIDWIFERY PLACE. Midwife Aotearoa New Zealand. 2025(117):44-5. | Wrong publication type (letter) |
| Shorten A, Shorten B. Independent midwifery care versus NHS care in the UK. BMJ. 2009;338:b2210. doi: https://dx.doi.org/10.1136/bmj.b2210. | Wrong publication type (editorial) |
| Sidery S, Bisits A, Spear V, Cummins A. Insights from a publicly funded homebirth program. Women Birth. 2025;38(1):101864. doi: doi:https://doi.org/10.1016/j.wombi.2024.101864. | Wrong comparison |
| Smith LA, Price N, Simonite V, Burns EE. Incidence of and risk factors for perineal trauma: a prospective observational study. BMC Pregnancy Childbirth. 2013;13:59. doi: https://dx.doi.org/10.1186/1471-2393-13-59. | Wrong population (not only women with normal pregnancies and expected normal births) |
| Snapp C, Stapleton SR, Wright J, Niemczyk NA, Jolles D. The Experience of Land and Water Birth Within the American Association of Birth Centers Perinatal Data Registry, 2012-2017. J Perinat Neonatal Nurs. 2020;34(1):16-26. doi: 10.1097/jpn.0000000000000450. | Wrong intervention (water birth) |
| Snowden JM, Tilden EL, Snyder J, Quigley B, Caughey AB, Cheng YW. Planned Out-of-Hospital Birth and Birth Outcomes. N Engl J Med. 2015;373(27):2642-53. doi: https://dx.doi.org/10.1056/NEJMsa1501738. | Wrong population (not only women with normal pregnancies and expected normal births) |
| Sprague AE, Sidney D, Darling EK, Van Wagner V, Soderstrom B, Rogers J, et al. Outcomes for the First Year of Ontario's Birth Center Demonstration Project. J Midwifery Womens Health. 2018;10:10. doi: https://dx.doi.org/10.1111/jmwh.12884. | Wrong population (not only women with normal pregnancies and expected normal births) |
| Stapleton SR, Osborne C, Illuzzi J. Outcomes of care in birth centers: demonstration of a durable model. J Midwifery Womens Health. 2013;58(1):3-14. doi: https://dx.doi.org/10.1111/jmwh.12003. | Wrong intervention (not possible to separate AMU/FMU) |
| Statens beredning för medicinsk utvärdering (SBU). Vetenskapligt underlag till Socialstyrelsens nationella riktlinjer för förlossningsvården: protokoll Stockholm: Statens beredning för medicinsk utvärdering (SBU); 2023a. Available from: https://www.sbu.se/371. | Wrong publication type (method description) |
| Statens beredning för medicinsk utvärdering (SBU). Vetenskapligt underlag för förstföderskor: Nationella riktlinjer för förlossningsvården (2023) Stockholm: Statens beredning för medicinsk utvärdering (SBU); 2023b. Available from: https://www.sbu.se/contentassets/cea2bfa2d5df4526b9ce0452dfd946dc/vetenskapligt-underlag-hemforlossning-forstfoderskor.pdf. | Wrong publication type (evidence summary, not a primary study) |
| Statens beredning för medicinsk utvärdering (SBU). Vetenskapligt underlag för omföderskor: Nationella riktlinjer för förlossningsvården (2023) Stockholm: Statens beredning för medicinsk utvärdering (SBU); 2023c. Available from: https://www.sbu.se/contentassets/cea2bfa2d5df4526b9ce0452dfd946dc/vetenskapligt-underlag-hemforlossning-omfoderskor.pdf. | Wrong publication type (evidence summary, not a primary study) |
| Stoll K, Kornelsen J. Midwifery care in rural and remote British Columbia: a retrospective cohort study of perinatal outcomes of rural parturient women with a midwife involved in their care, 2003 to 2008. J Midwifery Womens Health. 2014;59(1):60-6. doi: https://dx.doi.org/10.1111/jmwh.12137. | Wrong population (not only women with normal pregnancies and expected normal births) |
| Stolp I, Smit M, Luxemburg S, Akker T, Waard J, Roosmalen J, et al. Ambulance Transfer in Case of Postpartum Hemorrhage after Birth in Primary Midwifery Care in The Netherlands: A Prospective Cohort Study. Birth: Issues in Perinatal Care. 2015;42(3):227-34. doi: 10.1111/birt.12171. | Wrong population (only women who needed ambulance transfer) |
| Stramrood CA, Paarlberg KM, Huis In 't Veld EM, Berger LW, Vingerhoets AJ, Schultz WC, et al. Posttraumatic stress following childbirth in homelike- and hospital settings. J Psychosom Obstet Gynaecol. 2011;32(2):88-97. doi: https://dx.doi.org/10.3109/0167482X.2011.569801. | Wrong study design (survey study) |
| Strozik M, Wiciak H, Szarpak L, Wroblewski P, Smereka J. EMS Interventions during Planned Out-of-Hospital Births with a Midwife: A Retrospective Analysis over Four Years in the Polish Population. J Clin Med. 2023;12(24):15. doi: https://dx.doi.org/10.3390/jcm12247719. | Wrong population (pregnancy related emergency calls) |
| Suto M, Takehara K, Misago C, Matsui M. Prevalence of Perineal Lacerations in Women Giving Birth at Midwife-Led Birth Centers in Japan: A Retrospective Descriptive Study. J Midwifery Womens Health. 2015;60(4):419-27. doi: https://dx.doi.org/10.1111/jmwh.12324. | Wrong intervention (unclear intervention, mixed AMU/FMU) |
| Symon A, Winter C, Donnan PT, Kirkham M. Examining autonomy's boundaries: a follow-up review of perinatal mortality cases in UK independent midwifery. Birth. 2010;37(4):280-7. doi: https://dx.doi.org/10.1111/j.1523-536X.2010.00422.x. | Wrong study design |
| Symon A, Winter C, Inkster M, Donnan PT. Outcomes for births booked under an independent midwife and births in NHS maternity units: matched comparison study. BMJ. 2009;338:b2060. doi: https://dx.doi.org/10.1136/bmj.b2060. | Wrong population (not only women with normal pregnancies and expected normal births) |
| Symon AG, Paul J, Butchart M, Carr V, Dugard P. Self-rated "no-" and "low-" risk pregnancy: a comparison of outcomes for women in obstetric-led and midwife-led units in England. Birth. 2007;34(4):323-30. doi: https://dx.doi.org/10.1111/j.1523-536X.2007.00191.x. | Wrong outcome |
| Thornton P, McFarlin BL, Park C, Rankin K, Schorn M, Finnegan L, et al. Cesarean Outcomes in US Birth Centers and Collaborating Hospitals: A Cohort Comparison. J Midwifery Womens Health. 2017;62(1):40-8. doi: https://dx.doi.org/10.1111/jmwh.12553. | Wrong intervention (not ICM certified midwives) |
| Tucker J, McVicar A, Pitchforth E, Farmer J, Bryers H. Maternity care models in a remote and rural network: assessing clinical appropriateness and outcome indicators. Qual Saf Health Care. 2010;19(2):83-9. doi: https://dx.doi.org/10.1136/qshc.2008.028894. | Wrong population (not only women with normal pregnancies and expected normal births) |
| van der Kooy J, Birnie E, Denktas S, Steegers EAP, Bonsel GJ. Planned home compared with planned hospital births: Mode of delivery and Perinatal mortality rates, an observational study. BMC Pregnancy Childbirth. 2017;17. doi: https://dx.doi.org/10.1186/s12884-017-1348-y. | Wrong population (not only women with normal pregnancies and expected normal births) |
| van der Kooy J, de Graaf JP, Birnie DE, Denktas S, Steegers EA, Bonsel GJ. Different settings of place of midwife-led birth: evaluation of a midwife-led birth centre. Springerplus. 2016;5(1):786. doi: https://dx.doi.org/10.1186/s40064-016-2306-2. | Wrong population (not only women with normal pregnancies and expected normal births) |
| van der Kooy J, De Graaf JP, Kolder ZM, Witters KD, Fitzpatrick E, Duvekot JJ, et al. A newly developed scavenging system for administration of nitrous oxide during labour: safe occupational use. Acta Anaesthesiol Scand. 2012;56(7):920-5. doi: https://dx.doi.org/10.1111/j.1399-6576.2012.02668.x. | Wrong focus (No application) |
| van der Kooy J, Poeran J, de Graaf JP, Birnie E, Denktas S, Steegers EAP, et al. Planned home compared with planned hospital births in the Netherlands: intrapartum and early neonatal death in low-risk pregnancies. Obstet Gynecol. 2011;118(5):1037-46. doi: https://dx.doi.org/10.1097/AOG.0b013e3182319737. | Wrong intervention (not led by obstetricians) |
| van Haaren-ten Haken TM, Hendrix M, Smits LJ, Nieuwenhuijze MJ, Severens JL, de Vries RG, et al. The influence of preferred place of birth on the course of pregnancy and labor among healthy nulliparous women: a prospective cohort study. BMC Pregnancy Childbirth. 2015;15:33. doi: https://dx.doi.org/10.1186/s12884-015-0455-x. | Wrong study design (survey study) |
| Van Wagner V, Osepchook C, Harney E, Crosbie C, Tulugak M. Remote midwifery in Nunavik, Quebec, Canada: outcomes of perinatal care for the Inuulitsivik health centre, 2000-2007. Birth. 2012;39(3):230-7. doi: https://dx.doi.org/10.1111/j.1523-536X.2012.00552.x. | Wrong population (not only women with normal pregnancies and expected normal births) |
| Verdin M, Simon J, Gauchotte E, Morel O, Grandjean GA, Dap M. Obstetrical and neonatal outcomes of women intending to give birth in a Birth Center: A single-center retrospective study. Gynecologie Obstetrique Fertilite et Senologie. 2025;53(10):493-7. doi: https://dx.doi.org/10.1016/j.gofs.2025.04.003. | Wrong language (French) |
| Wallace J, Hoehn-Velasco L, Tilden E, Dowd BE, Calvin S, Jolles DR, et al. An alternative model of maternity care for low-risk birth: Maternal and neonatal outcomes utilizing the midwifery-based birth center model. Health Serv Res. 2024;59(1):e14222. doi: https://dx.doi.org/10.1111/1475-6773.14222. | Wrong population (not only women with normal pregnancies and expected normal births) |
| Wallis J. Planned home birth versus planned hospital birth. RCM Midwives. 2009;12(5):28. | Wrong publication type (research summary) |
| Walton C. The Birthplace in England Study: Methods, findings and evaluation. Br J Midwifery. 2012;20(1):22-7. | Wrong publication type (research summary) |
| Wasden SW, Chasen ST, Perlman JM, Illuzzi JL, Chervenak FA, Grunebaum A, et al. Planned home birth and the association with neonatal hypoxic ischemic encephalopathy. J Perinat Med. 2017;45(9):1055-60. doi: https://dx.doi.org/10.1515/jpm-2016-0292. | Wrong population (not only women with normal pregnancies and expected normal births) |
| Watterberg K. Planned home birth. Pediatr Rev. 2021;42:526-8. doi: https://dx.doi.org/10.1542/pir.2020-000364. | Wrong publication type (review) |
| Wax JR, Pinette MG, Cartin A, Blackstone J. Maternal and newborn morbidity by birth facility among selected United States 2006 low-risk births. Am J Obstet Gynecol. 2010;202(2):152.e1-5. doi: 10.1016/j.ajog.2009.09.037. | Wrong intervention (mixed attended/unattended) |
| Way EA, Carwile JL, Ziller EC, Ahrens KA. Out-of-hospital births and infant mortality in the United States: Effect measure modification by rural maternal residence. Paediatr Perinat Epidemiol. 2022;36(3):399-411. doi: https://dx.doi.org/10.1111/ppe.12862. | Wrong population (not only women with normal pregnancies and expected normal births) |
| Whittington JR, Rumpel JA, Shnaekel KL, Peeples SE, Magann EF, Burke BL. Alternative Birth Plans and Unintended Maternal and Neonatal Consequences: A Review of the Literature. Obstet Gynecol Surv. 2020;75:766-78. doi: https://dx.doi.org/10.1097/OGX.0000000000000849. | Wrong publication type (review) |
| Wiegerinck MM, Danhof NA, Van Kaam AH, Tamminga P, Mol BW. The validity of the variable "NICU admission" as an outcome measure for neonatal morbidity: a retrospective study. Acta Obstet Gynecol Scand. 2014;93(6):603-9. doi: https://dx.doi.org/10.1111/aogs.12384. | Wrong population (neonates admitted to NICU) |
| Wiegerinck MM, van der Goes BY, Ravelli AC, van der Post JA, Klinkert J, Brandenbarg J, et al. Intrapartum and neonatal mortality in primary midwife-led and secondary obstetrician-led care in the Amsterdam region of the Netherlands: A retrospective cohort study. Midwifery. 2015;31(12):1168-76. doi: https://dx.doi.org/10.1016/j.midw.2015.08.007. | Wrong population (not only women with normal pregnancies and expected normal births) |
| Wiegerinck MMJ, van der Goes BY, Ravelli ACJ, van der Post JAM, Buist FCD, Tamminga P, et al. Intrapartum and neonatal mortality among low-risk women in midwife-led versus obstetrician-led care in the Amsterdam region of the Netherlands: a propensity score matched study. BMJ Open. 2018;8(1):e018845. doi: https://dx.doi.org/10.1136/bmjopen-2017-018845. | Wrong intervention (mixed midwife-led/general practitioner-led) |

Abbreviations: AMU alongside midwifery unit, FMU freestanding midwifery unit, ICM International Confederation of Midwives, NICU neonatal intensive care unit

Supplementary Table S2. Risk of bias (ROBINS-I) at study level

| **Author, year** | **Directness** | **Risk of bias** | **Precision*** | | | | |
| --- | --- | --- | --- | --- | --- | --- | --- |
| Brocklehurst, 2011^3^ | + | ? | +^1^ | | | ?^2^ | |
| Davis, 2011^17^ | ? | - | +^3^ | | | -^4^ | |
| Davis, 2012^16^ | ? | - | + | | | | |
| Farry, 2019^18^ | - | ? | +^5^ | ?^6^ | | | -^7^ |
| Hermus, 2017^19^ | ? | ? | ?^8^ | | -^9^ | | |
| Hiraizumi, 2013^20^ | - | - | - | | | | |
| Homer, 2019^4^ | ? | ? | +^10^ | | ?^11^ | | |
| Lindgren, 2008a^21^ | + | ? | - | | | | |

The study by Scarf et al., 2019^22^ is only included for the outcome transfer to hospital and in subgroup analyses since otherwise overlapping with the study by Homer et al., 2019^4^ and has thus not been formally graded.

Risk of bias in non-randomised studies was assessed using domains based on the Risk Of Bias in Non-randomised Studies of Interventions (ROBINS-I) tool, as operationalised by the Swedish agency for health and technology assessment and assessment of social services (SBU).
Green=no problems, yellow=some problems, red=major problems 

^*^ within individual studies the precision varied for different outcomes, see number below for the outcomes:
^1^ intrapartum caesarean section, admission to neonatal ward or NICU, adverse infant events (asphyxia, Apgar score <7 at 5 min), perineal tear grade 3 or 4, postpartum haemorrhage requiring transfusion, maternal admission to ICU, admission to NICU ^2^ peri/neonatal mortality (within 7 or 28 days), neonatal mortality (within 7 or 28 days) , ^3^ for the outcome: intrapartum caesarean section 
^4^ peri/neonatal mortality (within 7 or 28 days), neonatal mortality (within 7 or 28 days) 
^5^ postpartum haemorrhage (>500 ml or >/≥1000 ml), admission to NICU
^6^ intrapartum caesarean section, adverse infant events (asphyxia, Apgar score <7 at 5 min) 
^7^ peri/neonatal mortality (within 7 or 28 days), neonatal mortality (within 7 or 28 days), maternal admission to ICU, maternal mortality 
^8^ postpartum haemorrhage (>500 ml or≥/>1000 ml), perineal tear grade 3 or 4 
^9^ admission to neonatal ward or NICU, postpartum haemorrhage requiring transfusion, peri/neonatal mortality (within 7 or 28 days), neonatal mortality (within 7 or 28 days), maternal mortality 
^10^ intrapartum caesarean section, postpartum haemorrhage requiring transfusion, maternal admission to ICU, admission to NICU
^11^ perineal tear grade 3 or 4, peri/neonatal mortality (within 7 or 28 days), neonatal mortality (within 7 or 28 days)

Supplementary Table S3. Certainty of evidence (GRADE) at outcome level

| **Outcome** | **Risk of bias*** | | **Consistency*** | | **Directness*** | | | | **Precision*** | | |
| --- | --- | --- | --- | --- | --- | --- | --- | --- | --- | --- | --- |
| Maternal mortality | ? ^4,5,8^) | | + ^4,5,8^ | | + ^8^ | ? ^5^ | | - ^4^ | - ^4,5,8^ | | |
| Maternal admission to ICU | ? ^1,4,7^ | | + ^1,4,7^ | | + ^1^ | ?  ^7^ | | - ^4^ | + ^1,7^ | - ^4^ | |
| Peri/neonatal mortality (within 7 or 28 days) | ? ^1,5,7,8^ | - ^2^ | + ^1,2,5,7,8^ | | + ^1,8^ | ? ^2,7^ | | - ^4^ | ? ^1,7^ | - ^2,4,8^ | |
| Neonatal mortality (within 7 or 28 days) | ? ^1,4,7,8^ | - ^2^ | + ^1,2,4,7,8^ | | + ^1,8^ | ? ^2,7^ | | - ^4^ | ? ^1,7^ | - ^2,4,8^ | |
| Apgar score <7 at 1 and/or 5 min, asphyxia | ? ^1,4^ | - ^6^ | + ^1,4,6^ | | + ^1^ | | - ^4,6^ | | + ^1^ | ? ^4^ | ^-^  ^6^ |
| Postpartum haemorrhage requiring transfusion | ? ^1,5,7^ | | + ^1,5,7^ | | + ^1^ | | ? ^5,7^ | | + ^1,7^ | ? ^5^ | |
| Perineal tear grade 3 or 4 | ? ^1,5,7^ | - ^6^ | ? ^1,5,6,7^ | | + ^1^ | ? ^5,7^ | | - ^6^ | + ^1^ | ? ^5,7^ | ^-^  ^6^ |
| Admission to neonatal ward or NICU | ? ^1,4,5,7^ | | + ^1,4,5,7^ | | + ^1^ | ? ^5,7^ | | - ^4^ | + ^1,4,7^ | - ^5^ | |
| Postpartum haemorrhage <500 ml or >/≥1000 ml | ? ^4,5^ | - ^3,6^ | + ^3,4,5^ | ^-^  ^6^ | ? ^3,5^ | | - ^4,6^ | | + ^3,4^ | ? ^5^ | ^-^  ^6^ |
| Intrapartum  caesarean section | ? ^1,4,7^ | - ^2^ | + ^1,2,4,7^ | | + ^1^ | ? ^2,7^ | | - ^4^ | + ^1,2,7^ | ? ^4^ | |

ICU=intensive care unit; NICU=neonatal intensive care unit

*Figures in table represent the eight included cohort studies. The superscripts refer to the references in the manuscript: 1=Brocklehurst et al., 2011^3^; 2=Davis et al., 2011^17^; 3=Davis et al., 2012^16^; 4=Farry et al., 2019^18^; 5=Hermus et al., 2017^19^; 6=Hiraizumi et al., 2013^20^; 7=Homer, 2019^4^; 8=Lindgren et al, 2008a^21^

Certainty of evidence was assessed using the Grading of Recommendations, Assessment, Development and Evaluation (GRADE) approach. Disagreements were resolved by consensus^15^.
Green=no problems, yellow=some problems, red=major problems. 

Supplementary Methods S1. Search strategy

**Database: Ovid MEDLINE(R) ALL (OvidSP)**

Date: 30 May 2024 
No. of results: 1,540

Search updated: 14 Mar 2025, 92 results

| **#** | **Searches** | **Results** |
| --- | --- | --- |
| 1 | exp *Home Childbirth/ | 2252 |
| 2 | (homebirth* or ((birth* or childbirth* or delivery or deliveries) adj3 (home or out-of-hospital* or non-hospital* or out-of-clinic* or non-clinic* or out-of-institution* or non-institution* or out-of-facilit* or non-facilit*))).ab,kf,ti. | 5349 |
| 3 | 1 or 2 | 6244 |
| 4 | exp Nurse Midwives/ | 7606 |
| 5 | midwi*.ab,kf,ti. | 31326 |
| 6 | planned.ab,kf,ti. | 107482 |
| 7 | 4 or 5 or 6 | 141082 |
| 8 | 3 and 7 | 1664 |
| 9 | exp *Birthing Centers/ | 668 |
| 10 | (birthcenter* or birthcentr* or birth-center* or birth-centr* or birthing center* or birthing centr* or community birth*).ab,kf,ti. | 1210 |
| 11 | 9 or 10 | 1524 |
| 12 | 4 or 5 | 34351 |
| 13 | 11 and 12 | 583 |
| 14 | (((midwi* adj3 unit) or (midwi* adj3 units) or (midwi* adj3 cent*) or (midwi* adj3 clinic) or (midwi* adj3 clinics)) and (birth* or childbirth* or delivery or deliveries)).ab,kf,ti. | 701 |
| 15 | 8 or 13 or 14 | 2566 |
| 16 | (comment or editorial or letter).pt. | 2250076 |
| 17 | 15 not 16 | 2431 |
| 18 | limit 17 to (danish or english or norwegian or swedish) | 2334 |
| 19 | limit 18 to yr="2005 -Current" | 1540 |

exp/ = term from the Medline controlled vocabulary, including terms found below this term in the MeSH hierarchy

adj3 = next to each other, in any order, up to 2 word(s) in between

.ab,kf,ti. = abstract, author keyword and title

.pt. = publication type

* = truncation of word for alternate endings

**Database: Embase 1974 to 2024 May 29 (OvidSP)**

Date: 30 May 2024

No of results: 1868 ref.

Search updated: 14 Mar 2025, 89 results

| **#** | **Searches** | **Results** |
| --- | --- | --- |
| 1 | exp *home delivery/ | 2169 |
| 2 | (homebirth* or ((birth* or childbirth* or delivery or deliveries) adj3 (home or out-of-hospital* or non-hospital* or out-of-clinic* or non-clinic* or out-of-institution* or non-institution* or out-of-facilit* or non-facilit*))).ab,kf,ti. | 6555 |
| 3 | 1 or 2 | 7305 |
| 4 | exp midwife/ | 36998 |
| 5 | "midwi*".ab,kf,ti. | 35224 |
| 6 | planned.ab,kf,ti. | 194789 |
| 7 | 4 or 5 or 6 | 241907 |
| 8 | 3 and 7 | 2310 |
| 9 | exp *maternity ward/ | 1057 |
| 10 | (birthcenter* or birthcentr* or birth-center* or birth-centr* or birthing center* or birthing centr* or community birth*).ab,kf,ti. | 1464 |
| 11 | 9 or 10 | 2308 |
| 12 | 4 or 5 | 48230 |
| 13 | 11 and 12 | 809 |
| 14 | (((midwi* adj3 unit) or (midwi* adj3 units) or (midwi* adj3 cent*) or (midwi* adj3 clinic) or (midwi* adj3 clinics)) and (birth* or childbirth* or delivery or deliveries)).ab,kf,ti. | 845 |
| 15 | 8 or 13 or 14 | 3532 |
| 16 | limit 15 to (article or article in press or conference paper or note or "review") | 2907 |
| 17 | limit 16 to (danish or english or norwegian or swedish) | 2749 |
| 18 | limit 17 to yr="2005 -Current" | 1868 |

exp/ = term from the Embase controlled vocabulary, including terms found below this term in the Emtree hierarchy

adj3 = next to each other, in any order, up to 2 word(s) in between

.ab,kf,ti. = abstract, author keyword and title

* = truncation of word for alternate endings

**Database: The Cochrane Library (Wiley)** 
Date: 30 May 2024
No of results: 221 ref

Search updated: 14 Mar 2025, 11 results

 Cochrane reviews: 10 
Cochrane protocols: 0
Trials: 211 
Editorials: 0 
Special collections: 0 
Clinical answers: 0

| **ID** | **Search** | **Hits** |
| --- | --- | --- |
| #1 | MeSH descriptor: [Home Childbirth] explode all trees | 61 |
| #2 | (homebirth* OR ((birth* OR childbirth* OR delivery OR deliveries) NEAR/2 (home OR "out of hospital" OR "out of hospitals" OR "non hospital" OR "non hospitals" OR "out of clinic" OR "out of clinics" OR "non clinic" OR "non clinics" OR "out of institution" OR "out of institutions" OR "non institution" OR "non institutions" OR "out of facility" OR "out of facilities" OR "non facility" OR "non facilities"))):ti,ab,kw (Word variations have been searched) | 609 |
| #3 | #1 OR #2 | 609 |
| #4 | MeSH descriptor: [Nurse Midwives] explode all trees | 137 |
| #5 | (midwi*):ti,ab,kw (Word variations have been searched) | 3306 |
| #6 | (planned):ti,ab,kw (Word variations have been searched) | 84663 |
| #7 | #4 OR #5 OR #6 | 87520 |
| #8 | #3 AND #7 | 148 |
| #9 | MeSH descriptor: [Birthing Centers] explode all trees | 20 |
| #10 | (birthcenter* OR birthcentr* OR "birth center" OR "birth centers" OR "birth centre" OR "birth centres" OR "birthing center" OR "birthing centers" OR "birthing centre" OR "birthing centres" OR "community birth" OR "community births" OR "community birthing"):ti,ab,kw (Word variations have been searched) | 100 |
| #11 | #9 OR #10 | 100 |
| #12 | #4 OR #5 | 3306 |
| #13 | #11 AND #12 | 28 |
| #14 | (((midwi* NEAR/2 unit) OR (midwi* NEAR/2 units) OR (midwi* NEAR/2 cent*) OR (midwi* NEAR/2 clinic) OR (midwi* NEAR/2 clinics)) AND (birth* OR childbirth* OR delivery OR deliveries)):ti,ab,kw (Word variations have been searched) | 212 |
| #15 | #8 OR #13 OR #14 | 377 |
| #16 | (clinicaltrials OR trialsearch):so | 507199 |
| #17 | (conference proceeding):pt | 242579 |
| #18 | #16 OR #17 | 749778 |
| #19 | #15 NOT #18 | 254 |
| Limit search to publication Year 2005- | | 221 |

MeSH descriptor: [] explode all trees = term from the MeSH controlled vocabulary, including terms found below this term in the hierarchy

NEAR/2 = Next to each other, in any order, up to 0 / 3 word(s) in between

:ti,ab,kw = title, abstract and author keywords

:pt = publication type

:so = source

* = truncation of word for alternate endings

**Database: CINAHL (EBSCOhost)**

Date: 30 May 2024

No. of results: 1939 ref.

Search updated: 14 Mar 2025, 71 results

| **#** | **Query** | **Limiters/Expanders** | **Results** |
| --- | --- | --- | --- |
| S17 | S8 OR S13 OR S14 | Limiters - Publication Date: 20050101-20241231; Language: Danish, English, Norwegian, Swedish  Expanders - Apply related words; Apply equivalent subjects  Search modes - Find all my search terms | 1,939 |
| S16 | S8 OR S13 OR S14 | Limiters - Language: Danish, English, Norwegian, Swedish  Expanders - Apply related words; Apply equivalent subjects  Search modes - Find all my search terms | 2,389 |
| S15 | S8 OR S13 OR S14 | Expanders - Apply related words; Apply equivalent subjects  Search modes - Find all my search terms | 2,463 |
| S14 | TI ( ((midwi* N2 unit) OR (midwi* N2 units) OR (midwi* N2 cent*) OR (midwi* N2 clinic) OR (midwi* N2 clinics)) and (birth* OR childbirth* OR delivery OR deliveries) ) OR AB ( ((midwi* N2 unit) OR (midwi* N2 units) OR (midwi* N2 cent*) OR (midwi* N2 clinic) OR (midwi* N2 clinics)) and (birth* OR childbirth* OR delivery OR deliveries) ) | Expanders - Apply related words; Apply equivalent subjects  Search modes - Find all my search terms | 595 |
| S13 | S11 AND S12 | Expanders - Apply related words; Apply equivalent subjects  Search modes - Find all my search terms | 644 |
| S12 | S4 OR S5 | Expanders - Apply related words; Apply equivalent subjects  Search modes - Find all my search terms | 41,823 |
| S11 | S9 OR S10 | Expanders - Apply related words; Apply equivalent subjects  Search modes - Find all my search terms | 1,451 |
| S10 | TI ( birthcenter* OR birthcentr* OR birth-center* OR birth-centr* OR "birthing center*" OR "birthing centr*" OR "community birth*" ) OR AB ( birthcenter* OR birthcentr* OR birth-center* OR birth-centr* OR "birthing center*" OR "birthing centr*" OR "community birth*" ) | Expanders - Apply related words; Apply equivalent subjects  Search modes - Find all my search terms | 1,076 |
| S9 | (MM "Alternative Birth Centers") | Expanders - Apply related words; Apply equivalent subjects  Search modes - Find all my search terms | 832 |
| S8 | S3 AND S7 | Expanders - Apply related words; Apply equivalent subjects  Search modes - Find all my search terms | 1,587 |
| S7 | S4 OR S5 OR S6 | Expanders - Apply related words; Apply equivalent subjects  Search modes - Find all my search terms | 74,369 |
| S6 | TI planned OR AB planned | Expanders - Apply related words; Apply equivalent subjects  Search modes - Find all my search terms | 33,157 |
| S5 | TI midwi* OR AB midwi* | Expanders - Apply related words; Apply equivalent subjects  Search modes - Find all my search terms | 34,059 |
| S4 | (MH "Midwives+") | Expanders - Apply related words; Apply equivalent subjects  Search modes - Find all my search terms | 17,020 |
| S3 | S1 OR S2 | Expanders - Apply related words; Apply equivalent subjects  Search modes - Find all my search terms | 4,913 |
| S2 | TI ( homebirth* OR ((birth* OR childbirth* OR delivery OR deliveries) N2 (home OR out-of-hospital OR non-hospital* OR out-of-clinic OR non-clinic* OR out-of-institution OR non-institution* OR out-of-facility OR non-facilit*)) ) OR AB ( homebirth* OR ((birth* OR childbirth* OR delivery OR deliveries) N2 (home OR out-of-hospital OR non-hospital* OR out-of-clinic OR non-clinic* OR out-of-institution OR non-institution* OR out-of-facility OR non-facilit*)) ) | Expanders - Apply related words; Apply equivalent subjects  Search modes - Find all my search terms | 4,005 |
| S1 | (MM "Home Childbirth") | Expanders - Apply related words; Apply equivalent subjects  Search modes - Find all my search terms | 2,275 |

MH "+" = term from the Cinahl controlled vocabulary, including terms found below this term in the Cinahl hierarchy

N2 = Next to each other, in any order, up to 2 word(s) in between

TI = title

AB = abstract

MM = word in major subject heading and core concept

* = truncation of word for alternate endings

The websites listed below were visited 30 May 2024 and 14 Mar 2025.

| **Source** | **Search terms / Browsing** | **No. of results** | **No. of relevant results** | **No. of relevant new results 14 Mar 2025** |
| --- | --- | --- | --- | --- |
| SBU:  Statens beredning för medicinsk och social utvärdering (the Swedish agency for health and technology assessment and assessment of social services)  [www.sbu.se](http://www.sbu.se/)    Limit to Rapporter [Reports] or Rapporter från andra HTA-organisationer [Reports from other HTA units]    Mark Visa även träffar äldre än 5 år [Also show results older than 5 years] | Hemförlossning  Hemförlossningar  Hemfödsel  Hemfödslar  Förlossning  Förlossningar  Förlossningsinrättning  Förlossningsinrättningar  Förlossningscenter  Förlossningsklinik  Förlossningskliniker  Barnmorskeledd  Barnmorskeledda  Barnmorskeassisterad  Barnmorskeassisterade | 2  2  0  0  59  59  0  0  0  9  9  0  0  1  1 | 1  1 (duplicate)  0  0  1 (duplicate)  1 (duplicate)  0  0  0  0  0  0  0  1 (duplicate)  1 (duplicate) | 2  2 (duplicates)  0  0  2 (duplicates)  2 (duplicates)  0  0  0  0  0  0  0  2 (duplicates)  2 (duplicates) |
| Folkehelseinstituttet (Norway)  https://www.fhi.no/publ/" \t "_blank"    Limit to publication type Systematisk oversikt [Systematic review] or Metodevurdering [Method evaluation] | Hjemmefødsel  Hjemmefødsler  Fødsel  Fødsler  Fødestue  Fødestuer  Fødeavdeling  Fødeavdelinger  Fødesentra  Fødesenter  Fødesentre  Fødselssentra  Fødselssenter  Fødselssentre  Mor-barn klinikk  Mor-barn klinikker  Jordmorstyrt  Jordmorstyrte  Jordmorassistert  Jordmorassisterte | 0  0  6  5  2  2  1  1  0  0  0  0  0  0  0  0  1  1  0  0 | 0  0  0  0  0  0  0  0  0  0  0  0  0  0  0  0  0  0  0  0 | 0  0  0  0  0  0  0  0  0  0  0  0  0  0  0  0  0  0  0  0 |
| Behandlingsrådet (Denmark)  <https://behandlingsraadet.dk/find-evalueringer-analyser> | Browsat | 0 | 0 | 0 |
| Nationale Kliniske Anbefalinger og Retningslinjer (Denmark)  <https://www.sst.dk/da/Fagperson/Retningslinjer-og-procedurer/NKA-og-NKR/NKR-og-NKA-efter-omraade> | Browsat | 0 | 0 | 0 |
| CAMTÖ: Centre for Assessment of Medical Technology in Örebro (Sweden)  https://www.regionorebrolan.se/sv/forskning/kontakt-och-organisation/hta-enheten-camto/ | Browsat | 0 | 0 | 0 |
| HTA Region Stockholm (Sweden)  <https://www.chis.regionstockholm.se/hta/rapporter/> | Browsat | 0 | 0 | 0 |
| Regional samverkansgrupp HTA (formerly Metodrådet) i Sydöstra sjukvårdsregionen (Sweden)  <https://sydostrasjukvardsregionen.se/samverkansgrupper/hta/genomforda-bedomningar/> | Browsat | 0 | 0 | 0 |
| HTA Syd (Sweden)  <https://vardgivare.skane.se/kompetens-utveckling/sakkunniggrupper/hta-skane/#110365> | Browsat | 0 | 0 | 0 |
| Medicinska rådet, Region Dalarna (30 May 2024) (Sweden)  <https://www.regiondalarna.se/plus/vard/utveckling-och-utbildning/kunskapsstyrning/medicinska-radet/>    Vetenskapliga rådet, Region Dalarna (14 Mar 2025) (Sweden)  <https://www.regiondalarna.se/plus/vard/utveckling-och-utbildning/kunskapsstyrning/vetenskapliga-radet/> | Browsat | 0 | 0 | 0 |
| International HTA database  <https://database.inahta.org/>    Limit to Year 2015-2024 | ((birth* OR childbirth* OR delivery OR deliveries) AND (home OR (out-of-hospital*) OR (non-hospital*) OR (out-of-clinic*) OR (non-clinic*) OR (out-of-institution*) OR (non-institution*) OR (out-of-facilit*) OR (non-facilit*)) AND (midwif* OR planned)) OR (homebirth* AND (midwif* OR planned)) OR ((birthcenter* OR birthcentr* OR (birth-center*) OR (birth-centr*) OR (birthing center*) OR (birthing centr*) OR (community birth*)) AND midwif*) OR (midwi* AND (unit OR units OR cent* OR clinic OR clinics) AND (birth* OR childbirth* OR delivery OR deliveries)) | 19 | 2 | 0 |

**Reference lists**

A comprehensive review of reference lists brought 12 new records.

Supplementary Figures S1a-S63b

Main analysis

Planned home/FMU births vs obstetric unit births: Figures S1a-S9b

Weighted risk difference with 95% CI: Figures S10-S18

Subgroup analysis according to parity

Outcomes in nulliparous women: Figures S19a-S27b

Outcomes in multiparous women: Figures S28a-S36b

Subgroup analysis according to place of birth

Outcomes in home births vs obstetric unit births: Figures S37a-S45b

Outcomes in FMU births vs obstetric unit births: Figures S46a-S54b

Sensitivity analysis

Sensitivity analysis of home/FMU births integrated in ordinary health care vs obstetric unit births: Figures S55-S63b

Outcomes critical for decision making:

*Maternal*

**Maternal admission to intensive care unit (ICU)**

Figure S1a. Odds ratio for maternal admission to intensive care unit (ICU)

Figure S1b. Adjusted* odds ratio for maternal admission to intensive care unit (ICU)

*Adjusted for: Brocklehurst: maternal age, parity, ethnic group, understanding English, BMI, index of multiple deprivation score, gestational age. Farry: maternal age, parity, smoking, ethnicity, BMI, socioeconomic status. Homer: maternal age, parity, country of birth, gestational age.

*Infant*

**Peri/neonatal mortality**

Figure S2a. Odds ratio for peri/neonatal mortality

Figure S2b. Adjusted* odds ratio for peri/neonatal mortality

*Adjusted for maternal age, parity, country of birth, and gestational age

Figure S3a. Odds ratio for neonatal mortality

Figure S3b. Adjusted* odds ratio for neonatal mortality

*Adjusted for maternal age, parity, country of birth, and gestational age

Outcomes important for decision making:

*Maternal*

Figure S4a. Odds ratio for postpartum haemorrhage requiring blood transfusion

Figure S4b. Adjusted* odds ratio for postpartum haemorrhage requiring transfusion

*Adjusted for: Brocklehurst: maternal age, parity, ethnic group, understanding English, BMI, index of multiple deprivation score, gestational age. Farry: maternal age, parity, smoking, ethnicity, BMI, socioeconomic status.

**Perineal tear grade 3-4**

Figure S5a. Odds ratio for perineal tear grade 3-4

Figure S5b. Adjusted* odds ratio for perineal tear grade 3-4

*Adjusted for: Brocklehurst: maternal age, parity, ethnic group, understanding English, BMI, index of multiple deprivation score, gestational age. Farry: maternal age, parity, smoking, ethnicity, BMI, socioeconomic status.

*Infant*

**Apgar <7 at 1 and/or 5 min**

Figure S6a. Odds ratio for Apgar <7 at 1 and/or 5 min

Figure S6b. Adjusted* odds ratio for Apgar <7 at 5 min

*Adjusted for: Brocklehurst: maternal age, parity, ethnic group, understanding English, BMI, index of multiple deprivation score, gestational age. Farry: maternal age, parity, smoking, ethnicity, BMI, socioeconomic status.

**Admission to neonatal intensive care unit (NICU)**

Figure S7a. Odds ratio for admission to NICU

Figure S7b. Adjusted* odds ratio for admission to NICU

*Adjusted for: Brocklehurst: maternal age, parity, ethnic group, understanding English, BMI, index of multiple deprivation score, gestational age. Farry: maternal age, parity, smoking, ethnicity, BMI, socioeconomic status. Homer: maternal age, parity, country of birth, gestational age.

Outcomes less important for decision making:

*Maternal*

**Postpartum haemorrhage**

Figure S8a. Odds ratio for postpartum haemorrhage

Figure S8b. Adjusted* odds ratio for postpartum haemorrhage

*Adjusted for maternal age, parity, smoking, ethnicity, BMI, and socioeconomic status

Figure S9a. Odds ratio for intrapartum caesarean section

Figure S9b. Adjusted* odds ratio for intrapartum caesarean section

*Adjusted for: Brocklehurst: maternal age, parity, ethnic group, understanding English, BMI, index of multiple deprivation score, gestational age. Farry: maternal age, parity, smoking, ethnicity, BMI, socioeconomic status. Homer: maternal age, parity, country of birth, gestational age.

Weighted risk difference (RD) with 95% confidence interval (CI) for planned home/FMU births vs obstetric unit births in women with a low-risk pregnancy

Critical for decision-making

*Maternal*

**Maternal admission to intensive care unit (ICU)**

Figure S10. Weighted RD (95 CI) for maternal admission to ICU in women with home/FMU births vs obstetric unit births

*Infant*

**Peri/neonatal mortality**

Figure S11. Weighted RD (95% CI) for peri/neonatal mortality for home/FMU births vs obstetric unit births

**Neonatal mortality**

Figure S12. Weighted RD (95% CI) for neonatal mortality home/FMU births vs obstetric unit births

Important for decision-making

*Maternal*

**Postpartum haemorrhage requiring transfusion**

Figure S13. Weighted RD (95% CI) for postpartum haemorrhage requiring blood transfusion for home/FMU births vs obstetric unit births

**Perineal tears grade 3-4**

Figure S14. Weighted RD (95% CI) for perineal tears grade 3-4 for home/FMU births vs obstetric unit births

*Infant*

**Apgar score <7 at 1 and/or 5 min**

Figure S15. Weighted RD (95% CI) for Apgar score < 1 and/or 5 min for infants in home/FMU births vs obstetric unit births

**Admission to neonatal intensive care unit (NICU)**

Figure S16. Weighted RD (95% CI) for admission to NICU for infants in home/FMU births vs obstetric unit births

Less important for decision-making

**Postpartum haemorrhage (>500 ml, ≥/>1000 ml)**

Figure S17. Weighted RD (95% CI) for postpartum haemorrhage for home/FMU births vs obstetric unit births

**Intrapartum caesarean section**

Figure S18. Weighted RD (95% CI) for intrapartum caesarean section in planned FMU births vs obstetric unit births

**Subgroup analyses according to parity**

Nulliparous women

Critical for decision-making

*Maternal*

**Maternal admission to intensive care unit (ICU) in nulliparous women**

Figure S19a. Odds ratio for maternal admission to ICU for home/FMU births vs obstetric unit births in nulliparous women

Figure S19b. Adjusted* odds ratio for maternal admission to ICU for home/FMU births vs obstetric unit births in nulliparous women

*Adjusted for maternal age, parity, ethnic group, marital or partner status, understanding English, BMI, index of multiple deprivation score, and gestational age.

*Infant*

**Peri/neonatal mortality in nulliparous women**

Figure S20a. Odds ratio for peri/neonatal mortality for home/FMU births vs obstetric unit births in nulliparous women

Figure S20b. Adjusted* odds ratio for peri/neonatal mortality home/FMU births vs obstetric unit births in nulliparous women

*Adjusted for maternal age, parity, country of birth, and gestational age.

**Neonatal mortality in nulliparous women**

Figure S21. Odds ratio for neonatal mortality home/FMU births vs obstetric unit births in nulliparous women

Important for decision-making

*Maternal*

**Postpartum haemorrhage requiring transfusion**

Figure S22a. Odds ratio for postpartum haemorrhage requiring transfusion for home/FMU births vs obstetric unit births in nulliparous women

Figure S22b. Adjusted odds ratio for postpartum haemorrhage requiring transfusion for home/FMU births vs obstetric unit births in nulliparous women

*Adjusted for maternal age, parity, ethnic group, marital or partner status, understanding English, BMI, index of multiple deprivation score, and gestational age

Figure S23a. Odds ratio for perineal tear grade 3-4 for home/FMU births vs obstetric unit births in nulliparous women

Figure S23b. Adjusted* odds ratio for perineal tear grade 3-4 for home/FMU births vs obstetric unit births in nulliparous women

*Adjusted for maternal age, parity, ethnic group, marital or partner status, understanding English, BMI, index of multiple deprivation score, and gestational age

*Infant*

**Apgar score <7 at 5 min**

Figure S24a. Odds ratio for Apgar score <7 at 5 min for home/FMU births vs obstetric unit births in nulliparous women

Figure S24b. Adjusted* odds ratio for Apgar score <7 at 5 min for home/FMU births vs obstetric unit births in nulliparous women

*Adjusted for maternal age, parity, ethnic group, marital or partner status, understanding English, BMI, index of multiple deprivation score, and gestational age.

**Admission to neonatal intensive care units (NICU)**

Figure S25a. Odds ratio for admission to NICU for infants of nulliparous women in home/FMU births vs obstetric unit births

Figure S25b. Adjusted* odds ratio for admission to neonatal intensive care units (NICU) for infants of nulliparous women in home/FMU births vs obstetric unit births

*Adjusted for maternal age, parity, ethnic group, marital or partner status, understanding English, BMI, index of multiple deprivation score, and gestational age

Less important for decision-making

*Maternal*

**Postpartum haemorrhage (≥1000 ml)**

Figure S26. Odds ratio for postpartum haemorrhage for home/FMU births vs obstetric unit births in nulliparous women

**Intrapartum caesarean section**

Figure S27a. Odds ratio for intrapartum caesarean section for home/FMU births vs obstetric unit births in nulliparous women

Figure S27b. Adjusted* odds ratio for intrapartum caesarean section for home/FMU births vs obstetric unit births in nulliparous women

*Adjusted for maternal age, parity, ethnic group, marital or partner status, understanding English, BMI, index of multiple deprivation score, and gestational age

Multiparous women

Critical for decision-making

*Maternal*

**Maternal admission to intensive care unit (ICU)**

Figure S28a. Odds ratio for maternal admission to ICU for home/FMU births vs obstetric unit births in multiparous women

Figure S28b. Adjusted* odds ratio for maternal admission to ICU for home/FMU births vs obstetric unit births in multiparous women

*Adjusted for maternal age, parity, ethnic group, marital or partner status, understanding English, BMI, index of multiple deprivation score, and gestational age

*Infant*

**Peri/neonatal mortality**

Figure S29a. Odds ratio for peri/neonatal mortality for home/FMU births vs obstetric unit births in multiparous women

Figure S29b. Adjusted* odds ratio for peri/neonatal mortality for home/FMU births vs obstetric unit births in multiparous women

*Adjusted for maternal age, parity, country of birth, and gestational age.

**Neonatal mortality**

Figure S30. Odds ratio for neonatal mortality for home/FMU births vs obstetric unit births in multiparous women

Important for decision-making

*Maternal*

**Postpartum haemorrhage requiring transfusion**

Figure S31a. Odds ratio for postpartum haemorrhage requiring transfusion for home/FMU births vs obstetric unit births in multiparous women

Figure S31b. Adjusted* odds ratio for postpartum haemorrhage requiring transfusion for home/FMU births vs obstetric unit births in multiparous women

*Adjusted for maternal age, parity, ethnic group, marital or partner status, understanding English, BMI, index of multiple deprivation score, and gestational age.

**Perineal tears grade 3-4**

Figure S32a. Odds ratio for perineal tears grade 3-4 for home/FMU births vs obstetric unit births in multiparous women

Figure S32b. Adjusted* odds ratio for perineal tears grade 3-4 for home/FMU births vs obstetric unit births in multiparous women

*Adjusted for maternal age, parity, ethnic group, marital or partner status, understanding English, BMI, index of multiple deprivation score, and gestational age.

*Infant*

**Apgar <7 at 5 min**

Figure S33a. Odds ratio for Apgar score <7 at 5 min for home/FMU births vs obstetric unit births in multiparous women

Figure S33b. Adjusted* odds ratio for Apgar score <7 at 5 min for home/FMU births vs obstetric unit births in multiparous women

*Adjusted for maternal age, parity, ethnic group, marital or partner status, understanding English, BMI, index of multiple deprivation score, and gestational age.

**Admittance to neonatal intensive care units (NICU)**

Figure S34a. Odds ratio for admission to NICU for infants of multiparous women in home/FMU births vs obstetric unit births

Figure S34b. Adjusted* odds ratio for admission to NICU for home/FMU births vs obstetric unit births in infants of multiparous women

*Adjusted for maternal age, parity, ethnic group, marital or partner status, understanding English, BMI, index of multiple deprivation score, and gestational age.

Less important for decision-making

*Maternal*

**Postpartum haemorrhage (≥1000 ml)**

Figure S35. Odds ratio for postpartum haemorrhage for home/FMU births vs obstetric unit births in multiparous women

**Intrapartum caesarean section**

Figure S36a. Odds ratio for intrapartum caesarean section for home/FMU births vs obstetric unit births in multiparous women

Figure S36b. Adjusted* odds ratio for intrapartum caesarean section for home/FMU births vs obstetric unit births in multiparous women

*Adjusted for maternal age, parity, ethnic group, marital or partner status, understanding English, BMI, index of multiple deprivation score, and gestational age.

**Subgroup analyses according to place of birth:**

Planned home births (HB) vs obstetric unit (OU) births

Critical for decision-making

*Maternal*

**Maternal admission to intensive care unit (ICU)**

Figure S37a. Odds ratio for maternal admission to ICU in planned home births vs obstetric unit births

Figure S37b. Adjusted* odds ratio for maternal admission to ICU in planned home births vs obstetric unit births

Adjusted for: Brocklehurst: maternal age, parity, ethnic group, marital or partner status, understanding English, BMI, index of multiple deprivation score, gestational age; Homer: maternal age, parity, country of birth, gestational age

*Infant*

**Peri/neonatal mortality**

Figure S38a. Odds ratio for peri/neonatal mortality in planned home births vs obstetric unit births

Figure S38b. Adjusted* odds ratio for peri/neonatal mortality in planned home births vs obstetric unit births

*Adjusted for maternal age, parity, country of birth, and gestational age

**Neonatal mortality**

Figure S39a. Odds ratio for neonatal mortality in planned home births vs obstetric unit births

Figure S39b. Adjusted* odds ratio for neonatal mortality in planned home births vs obstetric unit births

*Adjusted for maternal age, parity, country of birth, and gestational age

*Important for decision-making*

*Maternal*

**Postpartum haemorrhage requiring transfusion**

Figure S40a. Odds ratio for postpartum haemorrhage requiring transfusion in planned home births vs obstetric unit births

Figure S40b. Adjusted* odds ratio for postpartum haemorrhage requiring transfusion in planned home births vs obstetric unit births

*Adjusted for: Brocklehurst: maternal age, parity, ethnic group, marital or partner status, understanding English, BMI, index of multiple deprivation score, gestational age; Homer: maternal age, parity, country of birth, gestational age

**Perineal tears grade 3-4**

Figure S41a. Odds ratio for perineal tears grade 3-4 in planned home births vs obstetric unit births

Figure S41b. Adjusted* odds ratio for perineal tears grade 3-4 in planned home births vs obstetric unit births

*Adjusted for: Brocklehurst: maternal age, parity, ethnic group, marital or partner status, understanding English, BMI, index of multiple deprivation score, gestational age; Homer: maternal age, parity, country of birth, gestational age

*Infant*

**Apgar score <7 at 1 and/or 5 min**

Figure S42a. Odds ratio for Apgar score <7 at 1 and/or 5 min in planned home births vs obstetric unit births

Figure S42b. Adjusted* odds ratio for Apgar score <7 at 5 min in planned home births vs obstetric unit births

*Adjusted for maternal age, parity, ethnic group, marital or partner status, understanding English, BMI, index of multiple deprivation score, and gestational age

**Admission to neonatal intensive care units (NICU)**

Figure S43a. Odds ratio for admission to NICU for infants in planned home births vs obstetric unit births

Figure S43b. Adjusted* odds ratio for admission to NICU for infants in planned home births vs obstetric unit births

*Adjusted for: Brocklehurst: maternal age, parity, ethnic group, marital or partner status, understanding English, BMI, index of multiple deprivation score, gestational age; Homer: maternal age, parity, country of birth, gestational age

Less important for decision-making

**Postpartum haemorrhage (>500, >/≥1000 ml)**

Figure S44. Odds ratio for postpartum haemorrhage in planned home births vs obstetric unit births

**Intrapartum** **caesarean section**

Figure S45a. Odds ratio for intrapartum caesarean section in planned home births vs obstetric unit births

Figure S45b. Adjusted* odds ratio for intrapartum caesarean section in planned home births vs obstetric unit births

*Adjusted for: Brocklehurst: maternal age, parity, ethnic group, marital or partner status, understanding English, BMI, index of multiple deprivation score, gestational age; Homer: maternal age, parity, country of birth, gestational age

Births in FMU vs obstetric unit (OU) births

Critical for decision-making

*Maternal*

**Maternal admission to intensive care unit (ICU)**

Figure S46a. Odds ratio for maternal admission to ICU in planned FMU births vs obstetric unit births

Figure S46b. Adjusted* odds ratio for maternal admission to ICU in planned FMU births vs obstetric unit births

*Adjusted for: Brocklehurst: maternal age, parity, ethnic group, marital or partner status, understanding English, BMI, index of multiple deprivation score, gestational age; Farry: maternal age, parity, smoking, ethnic group, BMI, socioeconomic status

*Infant*

**Peri/neonatal mortality**

Figure S47. Odds ratio for peri/neonatal mortality in planned FMU births vs obstetric unit births

**Neonatal mortality**

Figure S48. Odds ratio for neonatal mortality in planned FMU births vs obstetric unit births

Important for decision-making

*Maternal*

**Postpartum haemorrhage requiring transfusion**

Figure S49a. Odds ratio for postpartum haemorrhage requiring transfusion in planned FMU births vs obstetric unit births

Figure S49b. Adjusted* odds ratio for postpartum haemorrhage requiring transfusion in planned FMU births vs obstetric unit births

*Adjusted for maternal age, parity, ethnic group, marital or partner status, understanding English, BMI, index of multiple deprivation score, and gestational age

**Perineal tears grade 3-4**

Figure S50a. Odds ratio for perineal tears grade 3-4 in planned FMU births vs obstetric unit births

Figure S50b. Adjusted* odds ratio for perineal tears grade 3-4 in planned FMU births vs obstetric unit births

*Adjusted for maternal age, parity, ethnic group, marital or partner status, understanding English, BMI, index of multiple deprivation score, and gestational age

*Infant*

**Apgar score <7 at 5 min**

Figure S51a. Odds ratio for Apgar score <7 at 5 min in planned FMU births vs obstetric unit births

Figure S51b. Adjusted* odds ratio for Apgar score <7 at 5 min in planned FMU births vs obstetric unit births

*Adjusted for: Brocklehurst: maternal age, parity, ethnic group, marital or partner status, understanding English, BMI, index of multiple deprivation score, gestational age; Farry: maternal age, parity, smoking, ethnic group, BMI, socioeconomic status

**Admission to neonatal intensive care units (NICU)**

Figure S52a. Odds ratio for admission to NICU for infants in planned FMU births vs obstetric unit births

Figure S52b. Adjusted* odds ratio for admission to admission to NICU in planned FMU births vs obstetric unit births

*Adjusted for: Brocklehurst: maternal age, parity, ethnic group, marital or partner status, understanding English, BMI, index of multiple deprivation score, gestational age; Farry: maternal age, parity, smoking, ethnic group, BMI, socioeconomic status

Less important for decision-making

**Postpartum haemorrhage**

Figure S53a. Odds ratio for postpartum haemorrhage in planned FMU births vs obstetric unit births

Figure S53b. Adjusted* odds ratio for postpartum haemorrhage in planned FMU births vs obstetric unit births

*Adjusted for maternal age, parity, smoking, ethnic group, BMI, and socioeconomic status

**Intrapartum caesarean section**

Figure S54a. Odds ratio for intrapartum caesarean section in planned FMU births vs obstetric unit births

Figure S54b. Adjusted* odds ratio for intrapartum caesarean section in planned FMU births vs obstetric unit births

*Adjusted for: Brocklehurst: maternal age, parity, ethnic group, marital or partner status, understanding English, BMI, index of multiple deprivation score, gestational age; Farry: maternal age, parity, smoking, ethnic group, BMI, socioeconomic status

Sensitivity analysis of births outside hospital integrated in ordinary health care vs obstetric unit births

Critical for decision-making

*Maternal*

**Maternal admission to intensive care unit (ICU)**

Figure S55. Odds ratio for maternal admission to ICU of births outside hospital integrated in ordinary health care vs obstetric unit births

*Infant*

**Peri/neonatal mortality**

Figure S56. Odds ratio for peri/neonatal mortality of births outside hospital integrated in ordinary health care vs obstetric unit births

**Neonatal mortality**

Figure S57. Odds ratio for neonatal mortality of births outside hospital integrated in ordinary health care vs obstetric unit births

Important for decision-making

*Maternal*

**Postpartum haemorrhage requiring transfusion**

Figure S58a. Odds ratio for postpartum haemorrhage requiring transfusion of births outside hospital integrated in ordinary health care vs obstetric unit births

Figure S58b. Adjusted* odds ratio for postpartum haemorrhage requiring transfusion of births outside hospital integrated in ordinary health care vs obstetric unit births

*Adjusted for maternal age, parity, ethnic group, marital or partner status, understanding English, BMI, index of multiple deprivation score, and gestational age

**Perineal tears grade 3-4**

Figure S59a. Odds ratio for perineal tear grade 3-4 of births outside hospital integrated in ordinary health care vs obstetric unit births

Figure S59b. Adjusted* odds ratio for perineal tear grade 3-4 of births outside hospital integrated in ordinary health care vs obstetric unit births

*Adjusted for maternal age, parity, ethnic group, marital or partner status, understanding English, BMI, index of multiple deprivation score, and gestational age

*Infant*

**Apgar score <7 at 5 min**

Figure S60a. Odds ratio for Apgar score <7 at 5 min of births outside hospital integrated in ordinary health care vs obstetric unit births

Figure S60b. Adjusted* odds ratio for Apgar score <7 at 5 min of births outside hospital integrated in ordinary health care vs obstetric unit births

*Adjusted for maternal age, parity, ethnic group, marital or partner status, understanding English, BMI, index of multiple deprivation score, and gestational age

**Admission to neonatal intensive care unit (NICU)**

Figure S61a. Odds ratio for admission to NICU of births outside hospital integrated in ordinary health care vs obstetric unit births

Figure S61b. Adjusted* odds ratio for admission to NICU of births outside hospital integrated in ordinary health care vs obstetric unit births

*Adjusted for maternal age, parity, ethnic group, marital or partner status, understanding English, BMI, index of multiple deprivation score, and gestational age

Less important for decision making

**Postpartum haemorrhage (≥1000 ml)**

Figure S62. Odds ratio for postpartum haemorrhage of births outside hospital integrated in ordinary health care vs obstetric unit births

**Intrapartum caesarean section**

Figure S63a. Odds ratio for intrapartum caesarean section of births outside hospital integrated in ordinary health care vs obstetric unit births

Figure S63b. Adjusted* odds ratio for intrapartum caesarean section of births outside hospital integrated in ordinary health care vs obstetric unit births

*Adjusted for maternal age, parity, ethnic group, marital or partner status, understanding English, BMI, index of multiple deprivation score, and gestational age

Supplementary Table S4. Selected studies on economic evaluations

| **Reference & reporting country** | **Costing perspective** | **Unit of reported costs** | **Cost of treatment (local currency)** | | | **Difference with obstetric unit costs** | | **Quality of cost estimation** |
| --- | --- | --- | --- | --- | --- | --- | --- | --- |
|  |  |  | **Home** | **FMU*** | **Obstetric unit** | **Home** | **FMU*** |  |
| Scarf et. al., 2021^45^, Australia | Public sector perspective | Mean | AUS$  4 748 | N.A.** | AUS$  5 463 | AUS$  715 or 13.1% lower | N.A.** | Good |
| Scarf et. al., 2020^46^, Australia | Health care perspective | Median (Range) | AUS$  2 150 (1 486-5 015) | AUS$  2 100 (1 555-3 808) | AUS$  2,097 (1 545-3 949) | AUS$  5 277 or 2.5% higher | *AUS$  3.2 or 0.15% lower (no difference) | Good |
| Schröder et. al., 2012^47^, England | Healthcare perspective | Mean (Standard Error) | £1 066 (8.9) | £14 35  (13.5) | £1 631 (10.1) | £565 or 34.4% lower | *£196 or 12% lower | Good |
| Joranger et. al., 2024^34^, Norway | Societal | Mean (Credible Interval) | Without on-call €1 872 (1 694 to 2 071)    With on-call  €5 531 (5 171-5 906) | N.A.** | €4 077 (3 575 to 4 615) | Without on-call €2 205 or 54% lower    With on-call  €1 454 or 35.7% higher | N.A.** | Good |
| Cicero et. al., 2022^44^, Italy | Third-payer (National Health Services) | Mean | €707 | €1 042 | €1 832 | €1 124 or 61.4% lower | €789 or 43.1% lower | Good |
